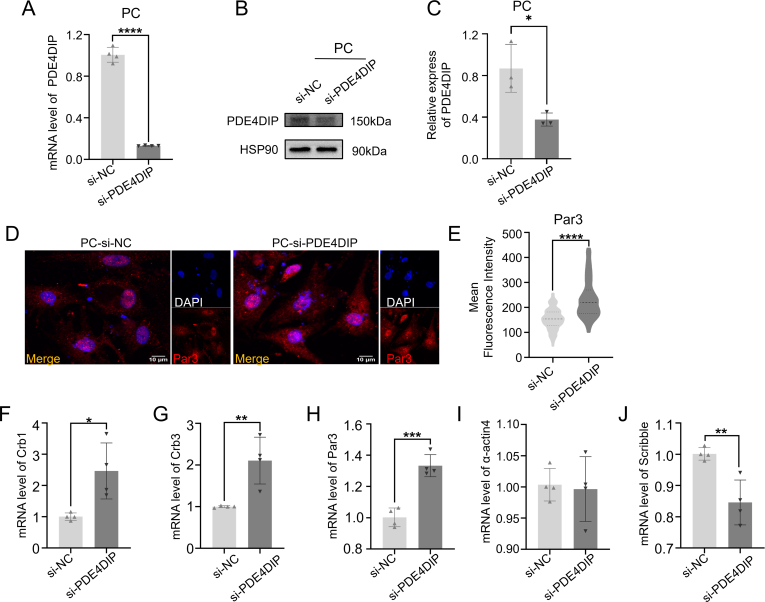# Dysfunction of PDE4DIP contributes to LVNC development by regulating cell polarity, skeleton, and energy metabolism via Rho-ROCK pathway

**DOI:** 10.1016/j.gendis.2025.101568

**Published:** 2025-02-21

**Authors:** Wuxia Gu, Hongyan Li, Wenjing Yuan, Xiaoqiong Fu, Rui Wang, Xiaohui Xu, Xuemei Liao, LingJuan Liu, Bo Pan, Jie Tian, Haixin Yuan, Yi Huang, Tiewei Lu

**Affiliations:** a Department of Cardiology, Children's Hospital of Chongqing Medical University, Ministry of Education Key Laboratory of Child Development and Disorders, China International Science and Technology Cooperation Base of Child Development and Critical Disorders, National Clinical Key Cardiovascular Specialty, Key Laboratory of Children's Important Organ Development and Diseases of Chongqing Municipal Health Commission, Chongqing Key Laboratory of Pediatrics, Chongqing 400014, China; b Department of Pediatric Research Institute, Children's Hospital of Chongqing Medical University, National Clinical Research Center for Child Health and Disorders, Ministry of Education Key Laboratory of Child Development and Disorders, Chongqing 400014, China; c Center for Novel Target and Therapeutic Intervention, Institute of Life Sciences, Chongqing Medical University, Chongqing 400016, China; d Department of Medicine, Washington University School of Medicine in St. Louis, St. Louis, MO 63110, USA

**Keywords:** Cell polarity, Cytoskeleton, Left ventricular non-compaction, Mitochondria, PDE4DIP, Rho-ROCK

## Abstract

Left ventricular non-compaction (LVNC), is a hereditary cardiomyopathy with limited treatments. Our previous study linked phosphodiesterase 4D interacting protein (PDE4DIP) to LVNC development. To explore the functional role of PDE4DIP activation in regulating cell polarity, skeleton, and energy metabolism, and to elucidate its mechanisms driving LVNC development, bioinformatics analysis was performed to compare its expression in LVNC patients and normal subjects. Overexpression and knockdown of PDE4DIP were constructed in H9C2 cells and neonatal Sprague–Dawley rat primary cardiomyocytes, respectively. Electron microscopy, MitoTracker-Green staining, and an ATP kit were employed to assess mitochondria's morphology and functional status. Real-time quantitative PCR, western blotting, and immunofluorescence assays were employed to detect the expression of cell polarity-, skeleton-, and Rho-ROCK signaling-related genes and proteins. Cell scratching and CCK-8 assays were employed to detect cell migration and proliferation abilities of H9C2, respectively. We found that PDE4DIP expression was increased in the LVNC-derived human-induced pluripotent stem cell-derived cardiomyocytes compared with normal subjects. Furthermore, overexpression of PDE4DIP induced cytoskeletal disorganization, decreased ATP content and cell migration, and increased cell proliferation and mitochondrial vacuolation. Moreover, the knockdown of PDE4DIP promoted cytoskeleton formation and contributed to increased ATP content and elevated cell migration. Mechanically, overexpression of PDE4DIP inhibited cell polarity-, skeleton-, and Rho-ROCK signaling-related genes and proteins, which could be increased by knockdown of PDE4DIP, suggesting that a critical regulation of PDE4DIP to Rho-ROCK pathway. This discovery suggests that PDE4DIP contributes to the development of LVNC by regulating cell polarity, skeleton, and energy metabolism through the Rho-ROCK pathway.

## Introduction

Left ventricular non-compaction (LVNC) is a genetically determined myocardial disorder, which mainly impacts the left ventricle, with coarse myofibrillar trabeculae protruding into the ventricular cavity and deep trabecular crypts as the main pathological features.^1^, ^2^, ^3^, ^4^ With a clinical incidence ranging from 0.05% to 0.3%, LVNC stands as the third most prevalent cardiomyopathy diagnosed in childhood,^5^, ^6^, ^7^, ^8^ typically manifesting with an average onset age between 90 days and 4 years after birth. About 20 % of the children present with clinical symptoms and signs, including arrhythmia, progressive cardiac insufficiency, and embolism, and in severe cases, sudden death may occur,^9^, ^10^, ^11^ but the clinical treatment is limited. Increasing evidence shows that myomere-, ion channel-, and mitochondria-related genes may be involved in the pathogenesis of LVNC, especially cytoskeleton-related genes that may contribute to cardiovascular diseases.^5^^,^^8^, ^9^, ^10^, ^11^, ^12^ However, the underlying mechanisms that drive the pathogenesis of LVNC remain largely elusive and inadequately comprehended. Therefore, identifying potential targets associated with the cytoskeleton during the development of LVNC could offer fresh perspectives and a deeper understanding of the mechanisms underlying its pathogenesis.

Phosphodiesterase 4D interacting protein (PDE4DIP), as a part of the AKAP protein complex,^13^^,^^14^ is mainly expressed in the vicinity of Z-discs and sarcoplasmic reticulum in cardiac and skeletal muscle ganglia.^15^ It has been shown that PDE4DIP plays a crucial role in cell polarity and skeleton by anchoring phosphodiesterase 4D (PDE4D) to the Golgi/centrosomal region of the cell, as well as in cell physiological activities by influencing cAMP synthesis and degradation.^16^ Most previous studies focused on chronic atrial fibrillation,^17^ dilated cardiomyopathy,^18^ and myeloproliferative disorder associated with eosinophilia.^19^ Of note, our previous studies showed aberrant PDE4DIP levels in 3 LVNC families,^20^ indicating that PDE4DIP may be associated with LVNC development. However, much less information is available on the functional role of PDE4DIP in LVNC pathogenesis.

Thus, this study aims to investigate the role of PDE4DIP in cell polarity, skeleton, and energy metabolism of LVNC, and to explore potential mechanisms underlying LVNC development.

## Materials and methods

### Subjects and whole-exon sequencing

Three families with clinical manifestations of LVNC were recruited from the Children's Hospital of Chongqing Medical University as previously described.^20^, ^21^, ^22^, ^23^, ^24^ Peripheral blood was collected from the three LVNC families for whole-exon sequencing (Wuhan KC seqhealth Co., Ltd., Wuhan, China) as described previously^20^ (Supplementary Material 2.1). All procedures have been reviewed and approved by the Medical Research Ethics Committee of Children's Hospital affiliated to Chongqing Medical University.

### Cell lines, reagents, and animal

Human-induced pluripotent stem cells (hiPSCs) and hiPSC-derived cardiomyocytes (hiPSC-CMs) were maintained as our previous reports^20^^,^^21^ (Supplementary Material 2.2; Fig. S1A). The neonatal Sprague–Dawley (SD) rats were purchased from the Animal Center of Chongqing Medical University (Chongqing, China). The primary myocardial cells of neonatal SD rats, recorded as PC, were extracted from the hearts of the neonatal SD rats within 3 days of birth and were cultured in DMEM/F-12 (Gibco, USA) supplemented with 10% fetal bovine serum (Sigma, USA) (Supplementary Material 2.2; Fig. S1B). Rat embryonic cardiomyocyte H9C2 cells were cultured in DMEM (Gibco, USA) supplemented with 10 % fetal bovine serum (Hyclone, USA) in an incubator containing 5% CO_2_ at 37 °C.^23^ All animal-related procedures were reviewed and approved by the Institutional Animal Care and Use Committee of the Affiliated Children's Hospital of Chongqing Medical University (IACUC Issue No: CHCMU-IACUC20231122013).

### Plasmids, small interferences (siRNA), and cell transfections

The PDE4DIP overexpression plasmid (P-PDE4DIP) and its negative control (P-NC) were purchased from GeneChem Co., China (Supplementary Material 1; Fig. S1C). The siRNA and GP-Transfect-Mate reagent were purchased from GenePharma (China) and included PDE4DIP-small interference (si-PDE4DIP) (target sequences from 5′ to 3′: CAGCAUCUCAGGCAUUCAUTT) and corresponding negative control small interference (si-NC) (target sequences from 5′ to 3′: UUCUCCGAACGUGUCACGUTT). For plasmids transfection, the cells transfected P-PDE4DIP and P-NC with Lipofectamine 3000 reagent (Thermo Fisher Science, USA) respectively. For siRNA transfection, the cells transfected si-PDE4DIP and si-NC with GP-Transfect-Mate reagent (GeneChem Co, China) respectively. PDE4DIP expression was detected by western blotting and quantitative PCR as previously described.^20^^,^^23^ All antibodies and the primer sequences involved can be seen in Table S1 and S2.

### RNA extraction and quantitative real-time PCR

Total RNA was extracted by using an RNA extraction kit (Bio Flux, China). A reverse transcription kit (Accurate Biology, China) was used for reverse transcription. Quantitative PCR was conducted on a real-time PCR system (Bio-Rad, USA) using SYBR Green (Accurate Biology). Data analysis was performed using the 2^−ΔΔCT^ method. GAPDH was used for normalization. The primer sequences involved can be found in Table S1.

### Protein extraction and western blotting

Total cellular proteins were extracted using RIPA lysis buffer (Beyotime, China) supplemented with protease inhibitor and phosphatase inhibitor (Beyotime, China). Protein extracts were quantified using an enhanced BCA protein assay kit (Beyotime, China). For western blotting, the proteins were transferred to 0.45 μM polyvinylidene fluoride (PVDF) membranes (Millipore, USA) after SDS-PAGE separation, and then were blocked with 5% nonfat milk at room temperature, followed by incubation with the diluted specific primary antibodies and the secondary antibodies. Finally, the PVDF membranes were visualized using a chemiluminescence reaction kit (Mishushengwu) on the ChemiDoc™ Touch Imaging System (Bio-Rad, USA). HSP90 was a loading control for total proteins. All antibodies in this study can be found in Table S2.

### Immunofluorescence staining

Indicated cells were fixed with 4% paraformaldehyde (Beyotime, China) for 20 min and then permeabilized using 0.5% Triton X-100 (Beyotime, China) for 10 min at room temperature. Indirect immunofluorescence staining was performed by incubation with the primary antibodies overnight at 4 °C, followed by incubation with the fluorescent secondary antibodies at 37 °C for 1 h. Nuclei were counter-stained with DAPI. The antibodies used in this study can be found in Table S2. The confocal dishes were examined with a confocal microscope (Nikon, Japan). Image analysis was performed with NIS Elements AR Analysis 5.20 software.

### Mito-Tracker staining

The cells cultured in confocal dishes were incubated with MitoTracker Green working solution (200 nM) in an incubator containing 5% CO_2_ at 37 °C for 25 min. After incubation, a confocal microscope was applied to acquire pictures. The fluorescence intensity was analyzed by NIS Elements AR Analysis 5.20 software and the morphological structure of mitochondria was analyzed by ImageJ software.

### Detection of ATP content

ATP level was measured using an ATP assay kit (Beyotime, China). Briefly, the cells were lysed by lysis solution and centrifuged to obtain the supernatant which was added to an opaque 96-well plate together with the ATP detection reagent. The RLU value was measured using a multifunction enzyme labeling instrument (Synergy H1, Bio Tek). The ATP concentration was normalized to the protein concentration.

### Cell counting kit-8 (CCK-8) assay

H9C2 cells were spread in a 96-well plate, CCK-8 solution (APExBIO) was added to each medium, and the conditions were maintained at 37 °C for 2 h. Optical density was measured by a plate reader (BioTek, USA) at a wavelength of 450 nm. This operation was repeated and cell proliferation was detected every 24 h. Every measurement was performed at least 3 times.

### Cell migration assays

H9C2 cells were spread in a 6-well plate, wounds were made using a 10 μL pipette tip, and images were taken at 0, 24, and 48 h after wounding. The distance migrated by the cell monolayer to close the wound area during this period was measured using Image J. Experiments were carried out in triplicate and repeated at least 3 times.

### Transmission electron microscopy

The cells were collected and fixed with 4% glutaraldehyde solution for 24 h and postfixed for 2 h with 1% osmium tetroxide at 4 °C. The cells were dehydrated with ethanol and methanol gradients and then embedded in epoxy resin. The samples were cut into thin (60 nm) sections and stained with citrate and uranyl acetate. Transmission electron microscope images were acquired at random locations throughout the samples and captured using a transmission electron microscope (H-7500).

### Statistical analysis

All data were analyzed and all graphs were generated by GraphPad Prism software (V8.3.0). An unpaired *t*-test was used in this study for two-group comparisons and one-way ANOVA was applied for multiple-group comparisons. All experiments were repeated at least 3 times in this study. *p* < 0.05 was considered statistically significant.

## Results

### The pathogenesis of LVNC involves cell polarity, skeleton, and energy metabolism

hiPSCs from LVNC patients and normal people were successfully constructed in our previous study,^20^^,^^21^ and it has been proved that they can be successfully induced into cardiomyocytes (hiPSC-CMs) (Video S1). The hiPSC-CMs were collected on the 30th day after induction. The transmission electron microscopy results showed that the number of micro-filaments decreased and the arrangement of micro-filaments was disordered in the LVNC group (Fig. 1A). The proportion of vacuolated mitochondria in cells increased and ATP content decreased (Fig. 1B, C). Confocal results showed that the expression of cytoskeleton proteins F-actin, vinculin, and polarity protein Par3 decreased (Fig. 1D–H). Gene ontology analysis was carried out on the sequencing results to find out the possible pathogenic mechanism of LVNC, and it was found that the enrichment areas included cell polarity, cytoskeleton, and energy metabolism.^22^ It was suggested that cell polarity, skeleton, and energy metabolism may be the pathogenesis of LVNC.

**Figure 1:**
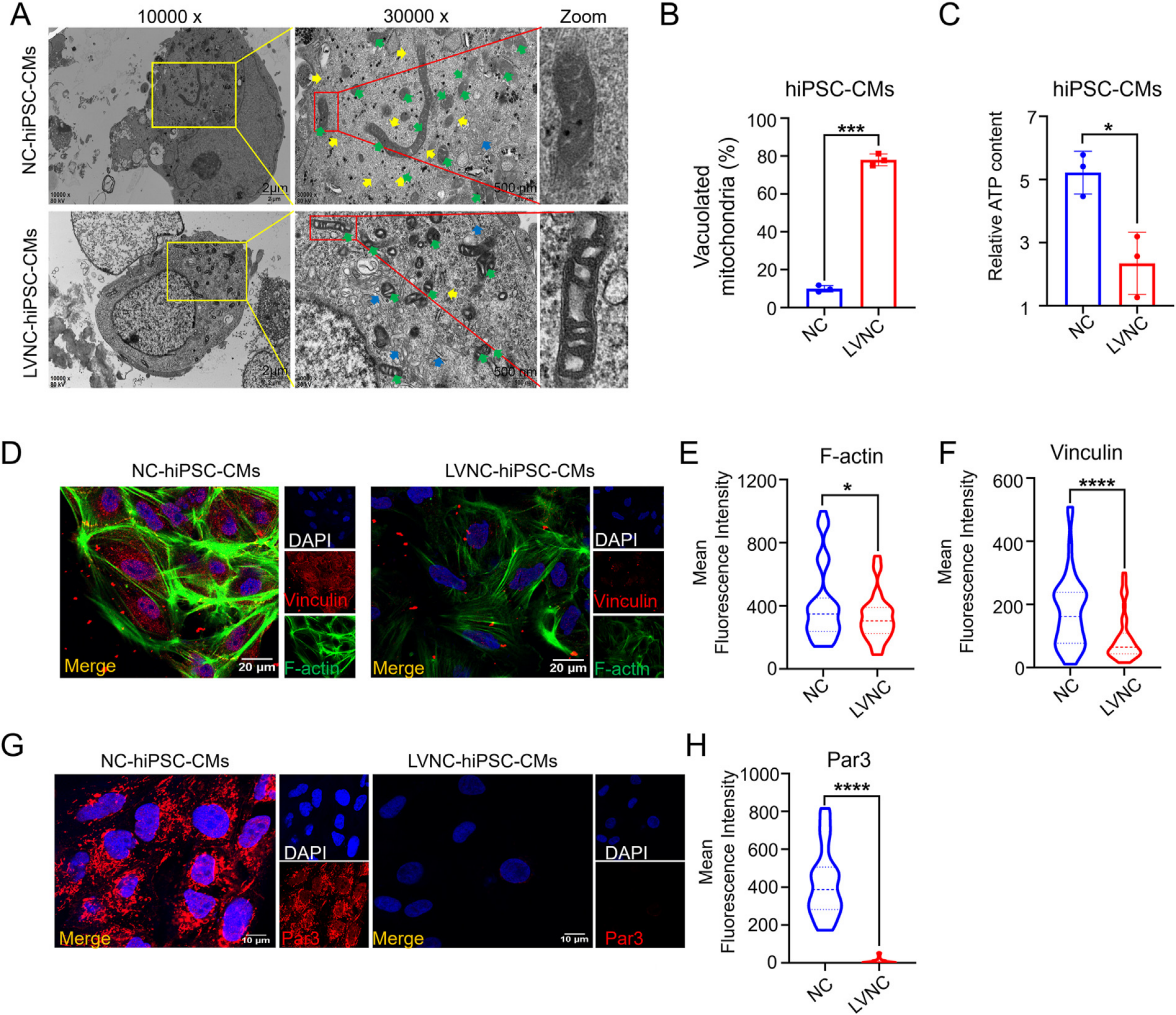
LVNC-hiPSC-CMs have abnormal skeleton, polarity, and mitochondria compared with the NC-hiPSC-CMs. **(A)** Representative transmission electron microscope images of hiPSC-CMs between the NC group and LVNC group. Myofibrils, yellow arrow; endoplasmic reticulum, blue arrow; mitochondria, green arrow. **(B, C)** The analysis of the proportion of the vacuolated mitochondria (*n* = 3 samples per group) and ATP content (nmol per mg protein) in hiPSC-CMs among the NC-hiPSC-CMs and LVNC-hiPSC-CMs groups (*n* = 3 samples per group). **(D**–**H)** Immunostaining of hiPSC-CMs for F-actin, vinculin, and Par3, and the analysis of the fluorescence intensity level (*n* = 30–80 cells per group). ∗∗∗∗*p* < 0.0001, ∗∗∗*p* < 0.001, and ∗*p* < 0.05 versus the NC group.

Supplementary video related to this article can be found at https://doi.org/10.1016/j.gendis.2025.101568

The following are the supplementary data related to this article:

VideoS1 2 VideoS1

VideoS2 3 VideoS2

VideoS3 4 VideoS3

### PDE4DIP is a new possible pathogenic gene of LVNC

Fourteen genes were co-expressed in three LVNC families by analyzing the possible pathogenic genes detected by whole-exon sequencing.^20^ Furthermore, the hiPSC-CMs were sequenced by transcriptome, and six differentially expressed genes were detected between the NC group and the LVNC patient group,^20^ among which the possible pathogenic gene related to cell polarity, skeleton, and energy metabolism was PDE4DIP. In the process of induction, cells at different time points were collected for quantitative PCR and western blotting experiments, and it was found that at the time point of 30 days, the expression of PDE4DIP in LVNC-hiPSC-CMs increased (Fig. 2A–E). Based on these results, PDE4DIP was considered a new pathogenic gene of LVNC. Combined with the cellular localization and physiological function of PDE4DIP (Fig. 2F–H), it was believed that PDE4DIP could lead to LVNC by affecting cell polarity, skeleton, and energy metabolism.

**Figure 2:**
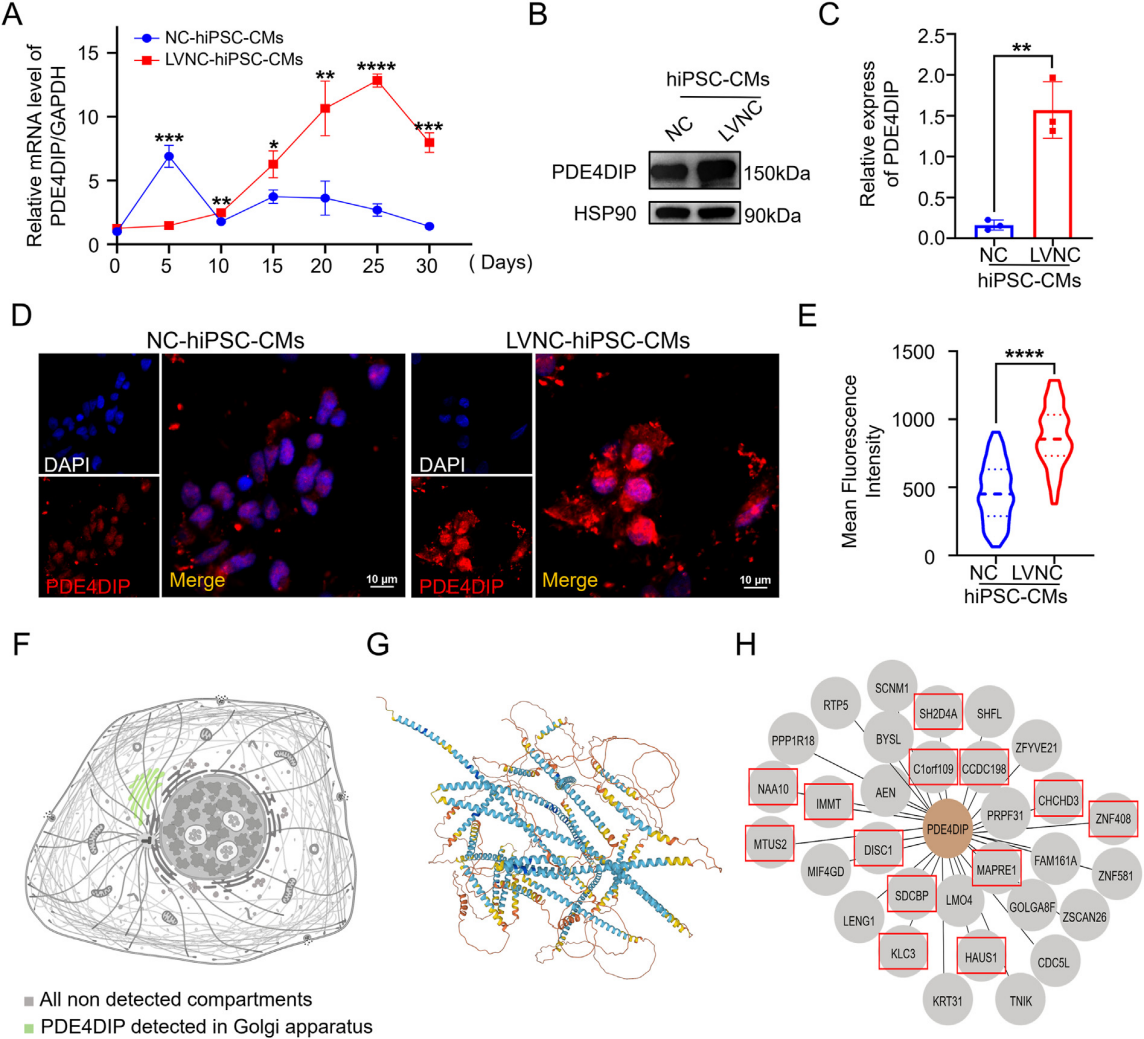
Changes of PDE4DIP expression after differentiation of hiPSCs into hiPSC-CMs. **(A)** Quantitative reverse transcription PCR analysis of PDE4DIP relative mRNA expression on days 0, 5, 10, 15, 20, 25, and 30 during hiPSC-CM differentiation. **(B, C)** Protein expression of PDE4DIP on the 30th day of inducement, and the analysis of the PDE4DIP protein expression (*n* = 3 samples per group). **(D, E)** Immunostaining of hiPSC-CMs for PDE4DIP (scale bar = 10 μm) among the NC-hiPSC-CMs and LVNC-hiPSC-CMs on the 30th day of inducement, and the analysis of PDE4DIP protein expression in 30th hiPS-CMs (*n* = 80–120 cells per group). **(F–H)** The schematic diagram shows the location of PDE4DIP in the Golgi apparatus (F), the supposed 3D structure map of PDE4DIP protein (G), and the interaction of PDE4DIP with other cytoskeleton-related genes in red boxes (H). ∗∗∗∗*p* < 0.0001, ∗∗∗*p* < 0.001, ∗∗*p* < 0.01, and ∗*p* < 0.05 versus the NC group.

### Overexpression of PDE4DIP caused cell polarity and skeleton disorganization and decreased energy metabolism in H9C2 cells

To fully understand the exact role of PDE4DIP in cell polarity, skeleton, and metabolism, H9C2 cells were transfected with plasmids to overexpress PDE4DIP, recorded as H9C2-P-NC and H9C2–P-PDE4DIP (Fig. S2A–C). In the LVNC disease model, the expression of polar protein decreased, the skeleton arrangement was disordered, and the proportion of vacuolated mitochondria increased. Therefore, compared with the P-NC group, the transmission electron microscopy results showed that myofilaments in the P-PDE4DIP group were decreased, the positional relationship was disordered, and the endoplasmic reticulum was swollen, suggesting that there was cytoskeleton abnormality in the P-PDE4DIP group (Fig. 3A). The morphology of mitochondria became round, the cristae were sparse, and the proportion of vacuolated mitochondria increased (Fig. 3B). Representative confocal microscope images showed mitochondrial morphology stained by MitoTracker-Green, and the results showed that the mitochondrial network and number of P-PDE4DIP were respectively disrupted and decreased compared with P-NC (Fig. 3C–E). In addition, after PDE4DIP overexpression, the ATP level, which represents the energy output of the cells, also decreased (Fig. 3F). The expression of cytoskeletal proteins vinculin, F-actin, and α/β-tubulin, and cellular polar proteins Par6 and Par3 (Fig. 3G–M; Fig. S2D, E) were observed with immunofluorescence. The fluorescence intensity was analyzed, and the results showed that the expression of skeleton proteins vinculin and F-actin decreased, α/β-tubulin increased, and polar protein Par6 decreased in the P-PDE4DIP group. Western blotting analysis revealed that the representative members of cytoskeleton and polarity proteins showed that in the P-PDE4DIP group, the expression of Scribble, Crb2, and vinculin decreased. In contrast, the expression of α/β-tubulin increased (Fig. 3N, O). The results of quantitative PCR showed that the expression of cytoskeleton-related genes Myh6 and α-actin4 decreased, and α-actin1 and α-tubulin increased; the expression of polarity-related genes Crb1, Crb2, and Par6b decreased, and Scribble, Crb3, and Par3 increased (Fig. 3P; Fig. S2F–J). The above results indicate that overexpression of PDE4DIP in H9C2 cells could lead to abnormal cytoskeleton, decreased metabolism, decreased expression of several cytoskeleton-related proteins and genes, and decreased expression of polarity-related proteins and genes. This is the same as the phenotype in the LVNC-derived hiPSCs-CMs.

**Figure 3:**
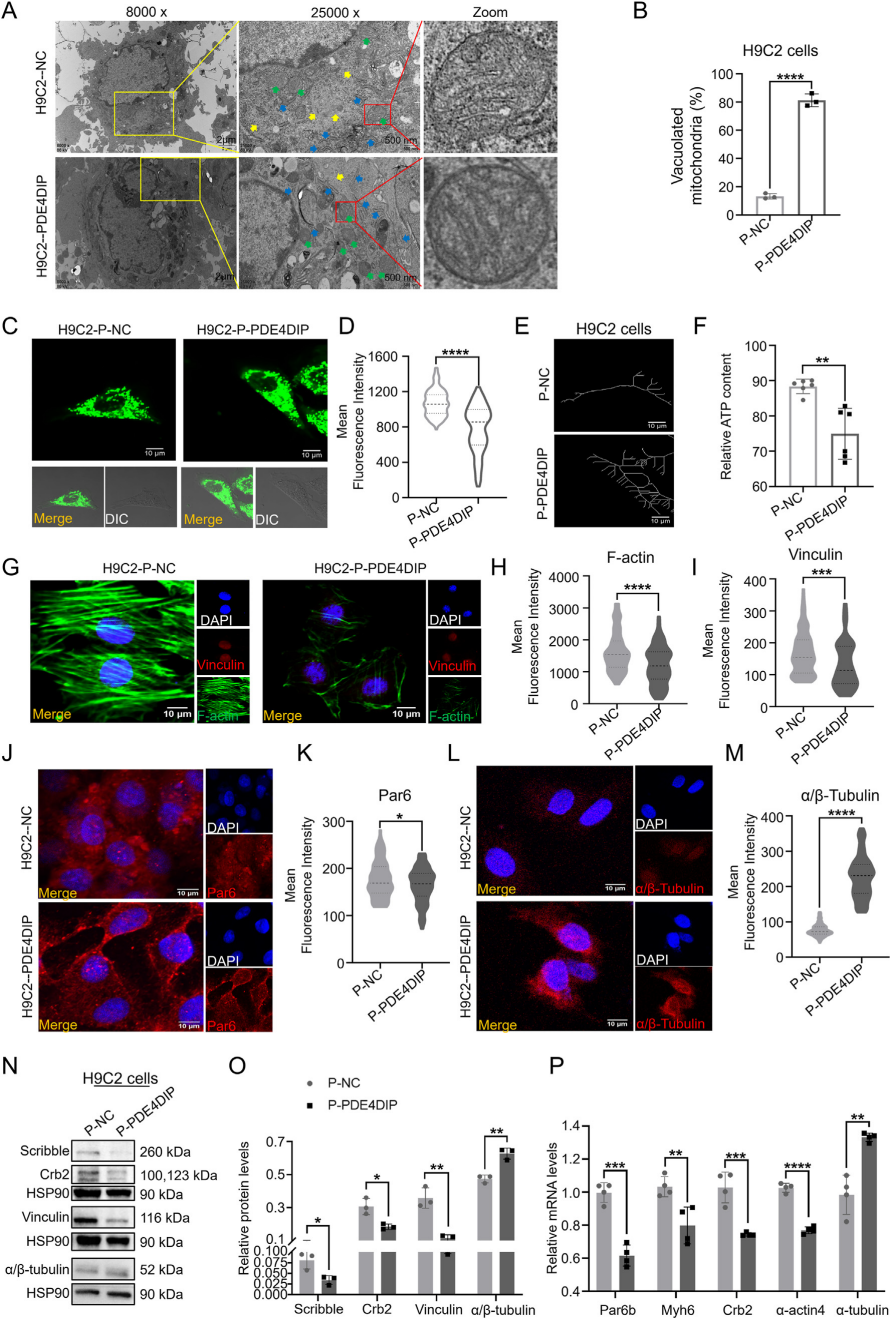
P-PDE4DIP has abnormal skeleton, polarity, and mitochondria in H9C2 cells compared with the P-NC. **(A)** Representative transmission electron microscope images of H9C2 cells between the P-NC group and P-PDE4DIP group. Myofibrils, yellow arrow; endoplasmic reticulum, blue arrow; mitochondria, green arrow. **(B)** The analysis of the proportion of the vacuolated mitochondria (*n* = 3 samples per group). **(C, D)** Representative confocal microscope images of mitochondrial morphology stained by MitoTracker-Green (scale bar = 10 μm) in H9C2 cells transfected with plasmids, and the analysis of the fluorescence intensity level (*n* = 80–120 cells per group). **(E)** Skeletonization of the mitochondrial network from MitoTracker staining by NIS analysis software, and ATP content (F) (nmol per mg protein) in H9C2 among the P-NC and P-PDE4DIP groups (*n* = 6). **(G**–**M)** Immunostaining of H9C2 transfected with plasmids for vinculin, F-actin (G–I), Par6 (J, K), and α/β-tubulin (L, M), and the analysis of the fluorescence intensity level (*n* = 80–120 cells per group). **(N, O)** Protein expression of H9C2 transfected with plasmids of Scribble, Crb2, α/β-tubulin, and vinculin, and the analysis of the protein expression (*n* = 3 samples per group). **(P)** Quantitative reverse transcription PCR was employed in H9C2 cells to assess the relative mRNA expression levels of cell polarity and cytoskeletal genes, including Crb2, Myh6, Par6b, α-actin4, and α-tubulin, in H9C2 cells, comparing the P-NC group with the P-PDE4DIP group. (*n* = 4 samples per group). ∗∗∗∗*p* < 0.0001, ∗∗∗*p* < 0.001, ∗∗*p* < 0.01, and ∗*p* < 0.05 versus the NC group.

### Overexpression of PDE4DIP caused cell polarity, skeleton disorganization, and decreased energy metabolism in primary cardiomyocytes of neonatal SD rats

H9C2 belongs to the embryonic period of SD rat cardiac cells, so whether it has the same effect in SD rat cardiac cells of different periods, therefore, primary cardiomyocytes (PC) were extracted from neonatal SD rats within 3 days of birth for subsequent verification (Fig. S1A). After overexpressed PDE4DIP in PC (Fig. S3A–C), the results of transmission electron microscopy (Fig. 4A) showed that compared with the P-NC group, the myofilaments in the P-PDE4DIP group were reduced. The positional relationship was chaotic, and the endoplasmic reticulum was swollen, suggesting that there was cytoskeleton abnormality in the P-PDE4DIP group. Mitochondrial morphology was rounded, cristae were sparse, the matrix of mitochondrial cristae was dense, and the proportion of vacuolated mitochondria was increased (Fig. 4B). Representative confocal MitoTracker-Green images showed that the mitochondrial network of P-PDE4DIP became deficient and more immature compared with P-NC (Fig. 4C–E). In addition, after PDE4DIP overexpression, the ATP level also decreased (Fig. 4F). The results of immunofluorescence showed that the fluorescence intensity of skeleton-related proteins vinculin, F-actin, and α/β-tubulin and of polar-related proteins Par6 and Par3 decreased in the P-PDE4DIP group (Fig. 4G–M; Fig. S3D, E). Western blotting experiment showed that in the P-PDE4DIP group, the expression of cytoskeleton and polarity proteins Scribble and vinculin decreased, while the expression of α/β-tubulin increased (Fig. 4N and O). The expression of cytoskeleton-related genes Myh6, α-actin1, and α-actin4 decreased, while the expression of α-tubulin increased; the expression of polarity-related genes Crb2, Crb3, Par6b, and Par3 decreased, while Crb1 and Scribble increased (Fig. 4P; Fig. S3F–J). These results show that overexpression of PDE4DIP in PC of newborn SD rats also leads to abnormal cytoskeleton, decreased metabolism, decreased expression of most cytoskeleton-related proteins and genes, and decreased expression of polarity-related proteins and genes. These results suggested that overexpression of PDE4DIP in PC led to the common phenotype in the LVNC-derived hiPSCs-CMs.

**Figure 4:**
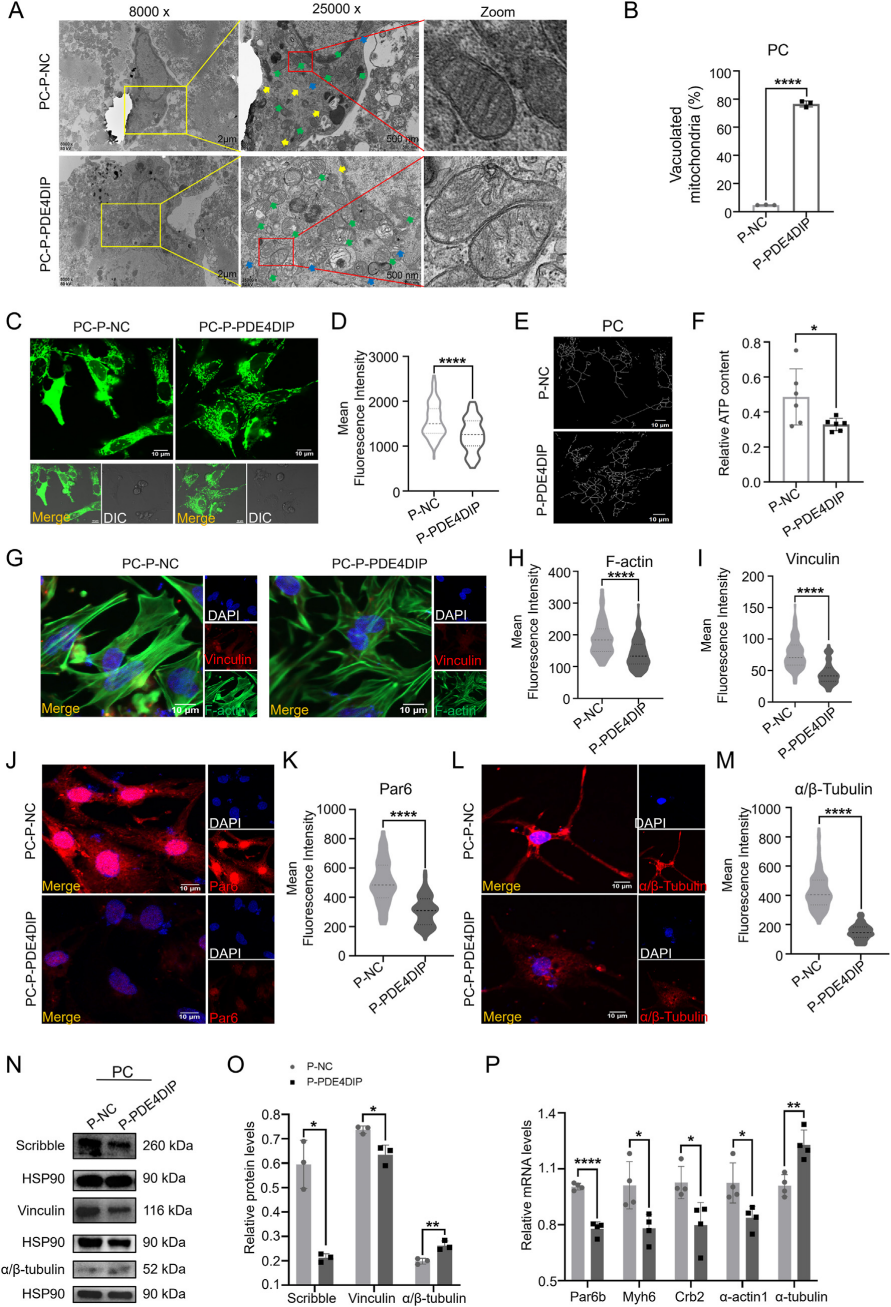
P-PDE4DIP has abnormal skeleton, polarity, and mitochondria in primary cardiomyocytes of neonatal Sprague–Dawley rats within 3 days of birth (PC), compared with the P-NC. **(A)** Representative transmission electron microscope images of primary cardiomyocytes between the P-NC group and P-PDE4DIP group. Myofibrils, yellow arrow; endoplasmic reticulum, blue arrow; mitochondria, green arrow. **(B)** The analysis of the proportion of the vacuolated mitochondria (*n* = 3 samples per group). **(C, D)** Representative confocal microscope images of mitochondrial morphology stained by MitoTracker-Green (scale bar = 10 μm) in PC transfected with plasmids, and the analysis of the fluorescence intensity level (*n* = 80–120 cells per group). **(E, F)** Skeletonization of the mitochondrial network from MitoTracker staining by NIS analysis software (E), and ATP content (nmol per mg protein) in PC among the P-NC and P-PDE4DIP groups (*n* = 6) (F). **(G**–**M)** Immunostaining of PC transfected with plasmids of vinculin, F-actin (G–I), Par6 (J, K), and α/β-tubulin (L, M), and the analysis of the fluorescence intensity level (*n* = 80–120 cells/group). **(N, O)** Protein expression of Scribble, vinculin, and α/β-tubulin, and the analysis of the protein expression (*n* = 3 samples/group). **(P)** Quantitative reverse transcription PCR was employed in PC to assess the relative mRNA expression levels of cell polarity and cytoskeletal genes, including Crb2, Myh6, Par6b, α-actin1, and α-tubulin, in H9C2 cells, comparing the P-NC group with the P-PDE4DIP group (*n* = 4 samples per group). ∗∗∗∗*p* < 0.0001, ∗∗*p* < 0.01, and ∗*p* < 0.05 versus the NC group.

### PDE4DIP knockdown improved cell polarity, skeleton, and energy release in H9C2 cells

**Figure 5:**
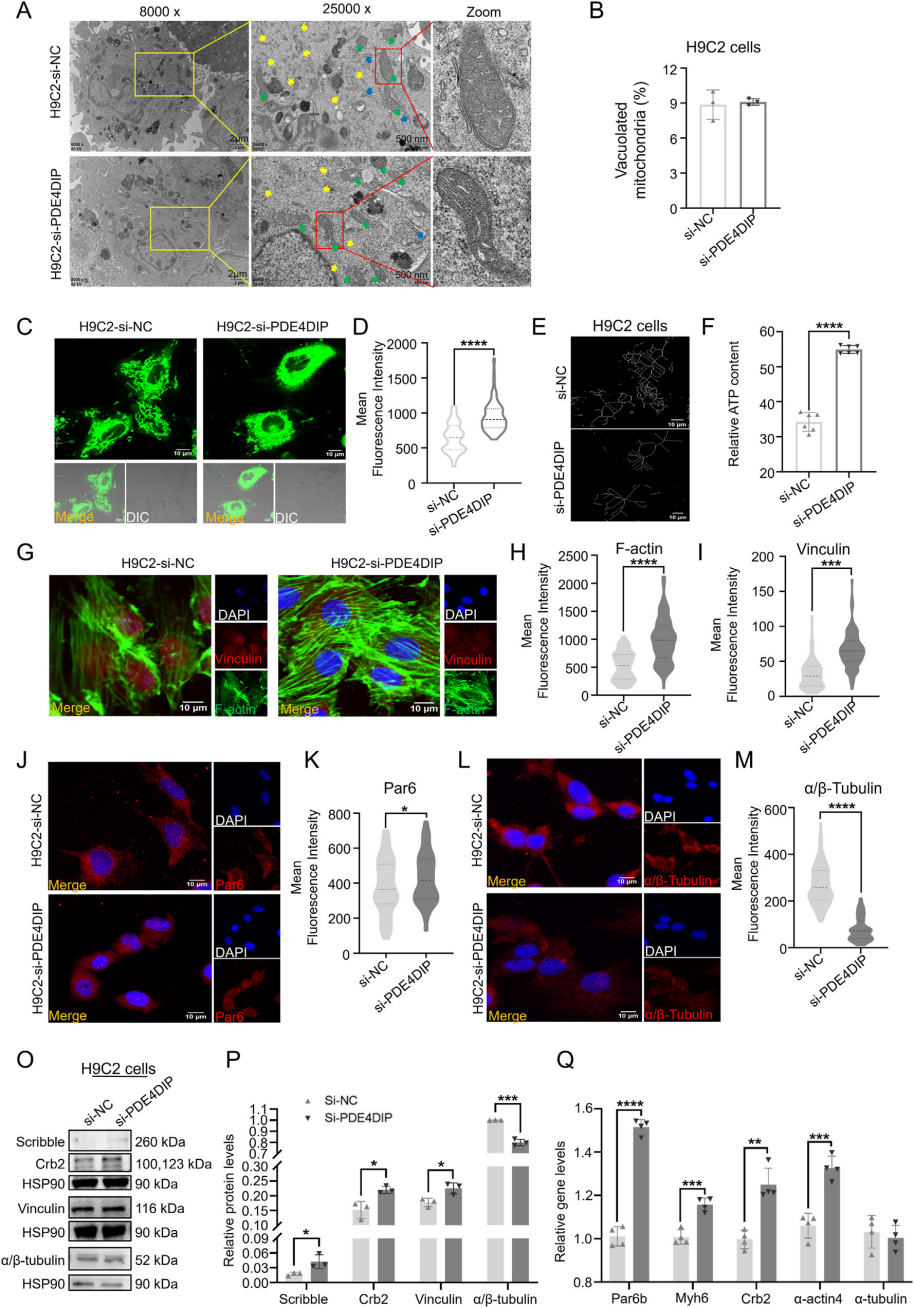
Changes in skeleton, polarity, and mitochondria after transfection of siRNA-PDE4DIP in H9C2 cells. **(A)** Representative transmission electron microscope images of H9C2 cells between the si-NC group and si-PDE4DIP group. Myofibrils, yellow arrow; endoplasmic reticulum, blue arrow; mitochondria, green arrow. **(B)** The analysis of the proportion of the vacuolated mitochondrial (*n* = 3 samples per group). **(C, D)** Representative confocal microscope images of mitochondrial morphology stained by MitoTracker-Green (scale bar = 10 μm) in H9C2 cells transfected with siRNA, and the analysis of the fluorescence intensity level (*n* = 80–120 cells per group). **(E, F)** Skeletonization of the mitochondrial network from MitoTracker staining by NIS analysis software (F), and ATP content (nmol per mg protein) in H9C2 among the si-NC and si-PDE4DIP groups (*n* = 6) (F). **(G**–**M)** Immunostaining of H9C2 transfected with plasmids of vinculin, F-actin (G–I), Par6 (J, K), and α/β-tubulin (L, M), and the analysis of the fluorescence intensity level (*n* = 80–120 cells per group). **(N, O)** Protein expression of PC transfected with siRNA of Scribble, Crb2, vinculin, and α/β-tubulin, and the analysis of the protein expression (*n* = 3 samples per group). **(P)** Quantitative reverse transcription PCR was employed in H9C2 cells to assess the relative mRNA expression levels of cell polarity and cytoskeletal genes, including Crb2, Myh6, Par6b, α-actin4, and α-tubulin, comparing the si-NC group and si-PDE4DIP group (*n* = 4 samples per group). ∗∗∗∗*p* < 0.0001, ∗∗∗*p* < 0.001, ∗∗*p* < 0.01, and ∗*p* < 0.05 versus the NC group.

In this study, the expression of PDE4DIP was knocked down by transfection of siRNA, recorded as si-NC and si-PDE4DIP (Figs. S4A–C). The results of transmission electron microscopy (Fig. 5A) showed that myofilaments in the si-PDE4DIP group increased and arranged more closely, and there was no obvious swelling of the endoplasmic reticulum. Mitochondria were regular in shape and the cristae were slender and arranged closely. The proportion of vacuolated mitochondria did not change significantly (Fig. 5B). Representative confocal MitoTracker-Green images showed that the mitochondrial network of si-PDE4DIP became abundant and mature compared with si-NC (Fig. 5C–E). In addition, after PDE4DIP knockdown, the ATP level also increased (Fig. 5F). The analysis of immunofluorescence showed that the fluorescence intensity of skeleton-related proteins vinculin and F-actin increased, α/β-tubulin decreased, and polar-related proteins Par6 and Par3 increased in the P-PDE4DIP group (Fig. 5G–M; Figs. S4D and E). Western blotting experiment showed that in the si-PDE4DIP group, the expression of cytoskeleton and polarity protein α/β-tubulin decreased, while the expression of Scribble, Crb2, and vinculin increased (Fig. 5N, O). The quantitative PCR results showed that the expression of cytoskeleton-related genes Myh6, α-actin1, and α-actin4 increased, α-tubulin decreased, the expression of polarity-related genes Crb1, Crb2, Crb3, and Par6b increased, and the expression of Par3 and Scribble decreased (Fig. 5P; Fig. S4F–J).

### PDE4DIP knockdown improved cell polarity, skeleton, and energy release in primary cardiomyocytes of neonatal SD rats

**Figure 6:**
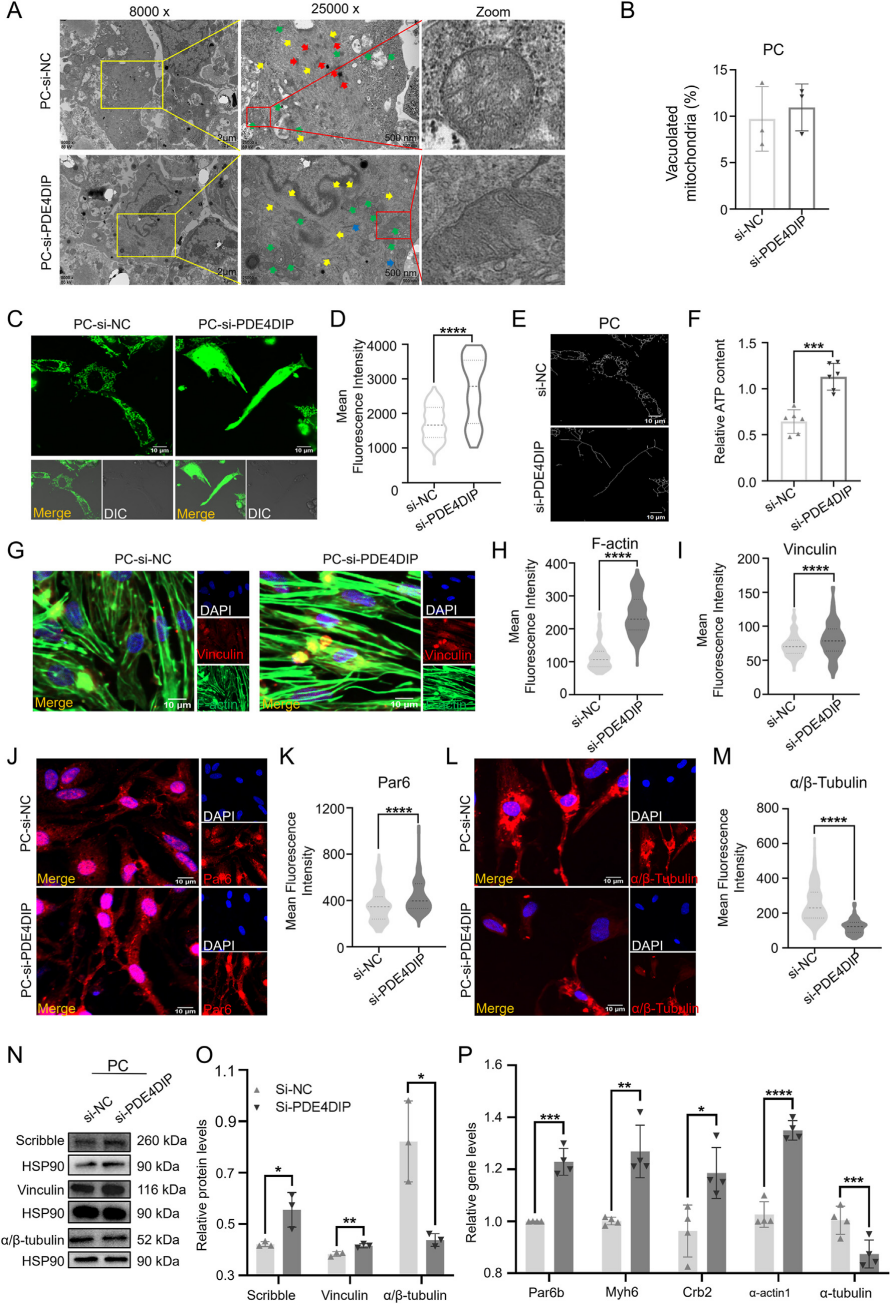
Changes in skeleton, polarity, and mitochondria after transfection of siRNA-PDE4DIP in the primary cardiomyocyte (PC) of neonatal Sprague–Dawley rats within 3 days of birth. **(A)** Representative transmission electron microscope images of primary cardiomyocytes between the si-NC group and si-PDE4DIP group. Myofibrils, yellow arrow; Z-disks, red arrow; endoplasmic reticulum, blue arrow; mitochondria, green arrow. **(B)** The analysis of the proportion of the vacuolated mitochondria (*n* = 3 samples per group). **(C, D)** Representative confocal microscope images of mitochondrial morphology stained by MitoTracker-Green (scale bar = 10 μm) in PC transfected with siRNA, and the analysis of the fluorescence intensity level (*n* = 80–120 cells per group). **(E, F)** Skeletonization of the mitochondrial network from MitoTracker staining by NIS analysis software (E), and ATP content (nmol per mg protein) in PC among the si-NC and si-PDE4DIP groups (*n* = 6) (F). **(G**–**M)** Immunostaining of PC transfected with siRNA for vinculin, F-actin (G–I), Par6 (J, K), and α/β-tubulin (L, M), and the analysis of the fluorescence intensity level (*n* = 80–120 cells per group). **(N, O)** Protein expression of PC transfected with siRNA of Scribble and vinculin, and the analysis of the protein expression (*n* = 3 samples per group). **(P)** Quantitative reverse transcription PCR was employed to assess the relative mRNA expression levels of cell polarity and cytoskeletal genes, including Crb2, Myh6, Par6b, α-actin1, and α-tubulin between the P-NC group and P-PDE4DIP group (*n* = 4 samples per group). ∗∗∗∗*p* < 0.0001, ∗∗∗*p* < 0.001, ∗∗*p* < 0.01, and ∗*p* < 0.05 versus the NC group.

After knocking down the expression of PDE4DIP in PC (Fig. S5A–C), the results of transmission electron microscopy (Fig. 6A) showed that the myofilaments in si-PDE4DIP group increased and arranged more closely, and *Z* bands formed sarcomere, without obvious swelling of the endoplasmic reticulum. The morphology of mitochondria was regular, the cristae were slender and arranged closely, and the proportion of vacuolated mitochondria did not change significantly (Fig. 6B). Representative confocal MitoTracker-Green images showed that the mitochondrial network of si-PDE4DIP became abundant and maturer (Fig. 6C–E), and the ATP level also increased (Fig. 6F). The fluorescence intensity of immunofluorescence was analyzed, and the results showed that the expression of skeleton proteins vinculin, F-actin, and polar proteins Par6 and Par3 were increased in the si-PDE4DIP group. At the same time, α/β-tubulin decreased (Fig. 6G–M; Fig. S5D, E). Western blotting experiment showed that the expression of cytoskeleton and polarity proteins Scribble and vinculin increased in the si-PDE4DIP group, while α/β-tubulin decreased (Fig. 6N, O). The results of quantitative PCR showed that the expression of cytoskeleton-related genes Myh6, α-actin1, and α-actin4 increased, while the expression of α-tubulin and Scribble decreased. The expression of polarity-related genes Crb1, Crb2, Crb3, Par6b, and Par3 increased, while Scribble decreased (Fig. 6P; Fig. S5F–J). The above results are consistent with the results of siRNA-PDE4DIP transfection in H9C2. It shows that the knockdown of PDE4DIP expression has the same effects on rat cardiomyocytes in different periods.

### PDE4DIP affected cell migration and proliferation in H9C2

**Figure 7:**
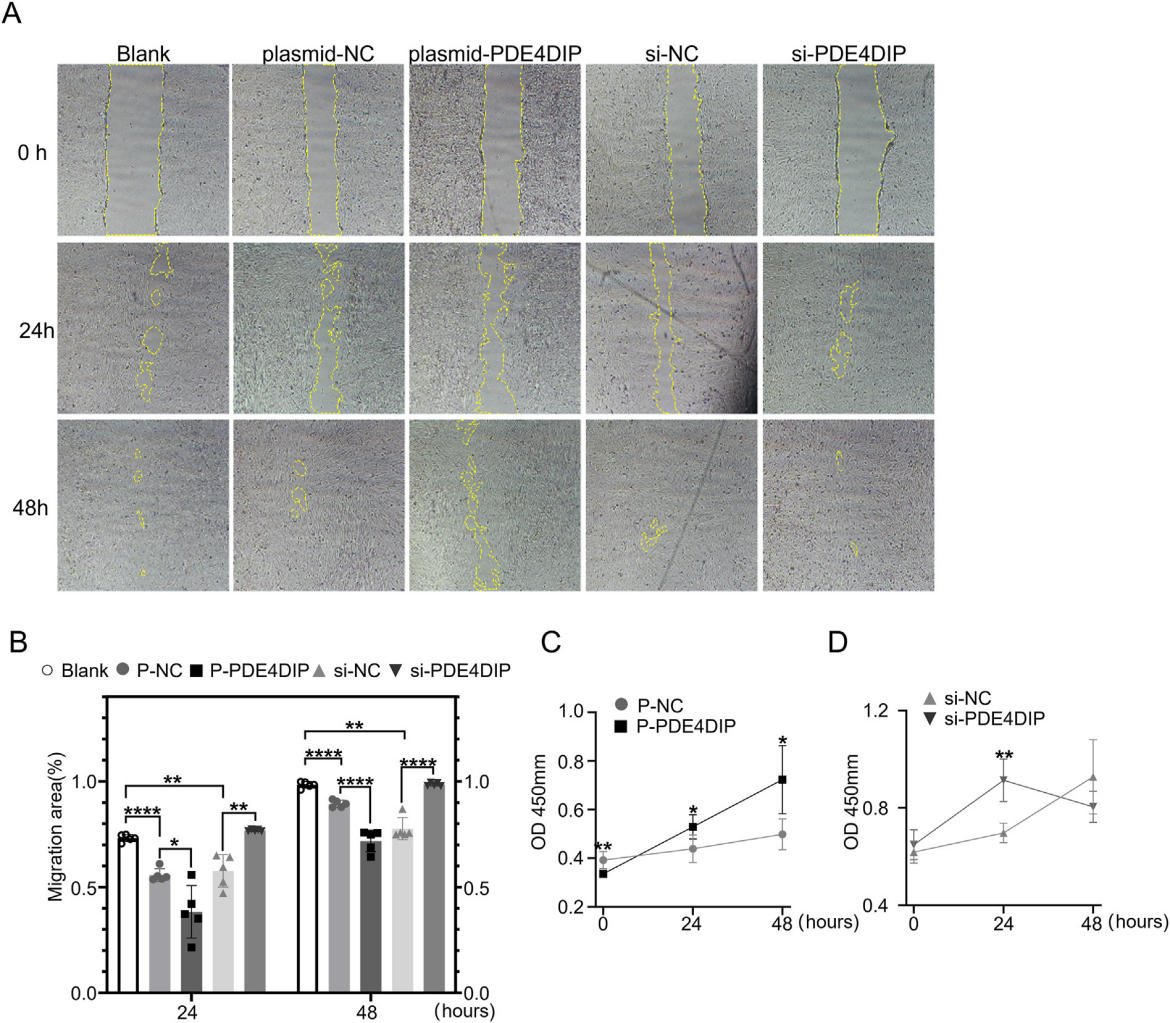
Changes of H9C2 cell migration and proliferation after transfection of plasmid-PDE4DIP and siRNA-PDE4DIP. **(A)** H9C2 cell migration in scratch wound experiment. **(B)** The analysis of the proportion of migration area (%) (*n* = 5 samples per group). **(C, D)** The H9C2 cells transfected with plasmid-NC, plasmid-PDE4DIP, siRNA-NC, and siRNA-PDE4DIP were investigated by the CCK-8 assay (*n* = 5). ∗∗∗∗*p* < 0.0001, ∗∗*p* < 0.01, and ∗*p* < 0.05 versus the NC group.

To investigate the role of PDE4DIP in migration and proliferation, we evaluated the effect of PDE4DIP overexpression and knockdown on migration and proliferation in H9C2 cells. As shown in Figure 7A and B, at 24 h and 48 h, the migration ability of P-PDE4DIP is weaker than P-NC group, and that of the si-PDEDIP is stronger than the si-NC group, indicating that the overexpression of PDE4DIP restrained cell migration, while knockdown of PDE4DIP promoted cell migration in H9C2 cells. The cell migration ability is related to the skeleton protein vinculin, a cytoskeleton protein associated with cell–cell and cell-matrix junctions.^25^^,^^26^ This is consistent with the previous confocal detection and western blotting experimental results. Secondly, CCK-8 was used to detect the proliferation of H9C2 transfected with plasmid and siRNA. The results showed that at 24 h, the proliferation ability of the P-PDE4DIP group was stronger than the P-NC group, and that of the si-PDE4DIP group was stronger than the si-NC group. At 48 h, the proliferation ability of the P-PDE4DIP group was stronger than the P-NC group, and that of the si-PDE4DIP group was weaker than the si-nc group (Fig. 7C, D).

### Rho-ROCK pathway is involved in PDE4DIP-affected cell polarity, cytoskeleton, and energy metabolism

**Figure 8:**
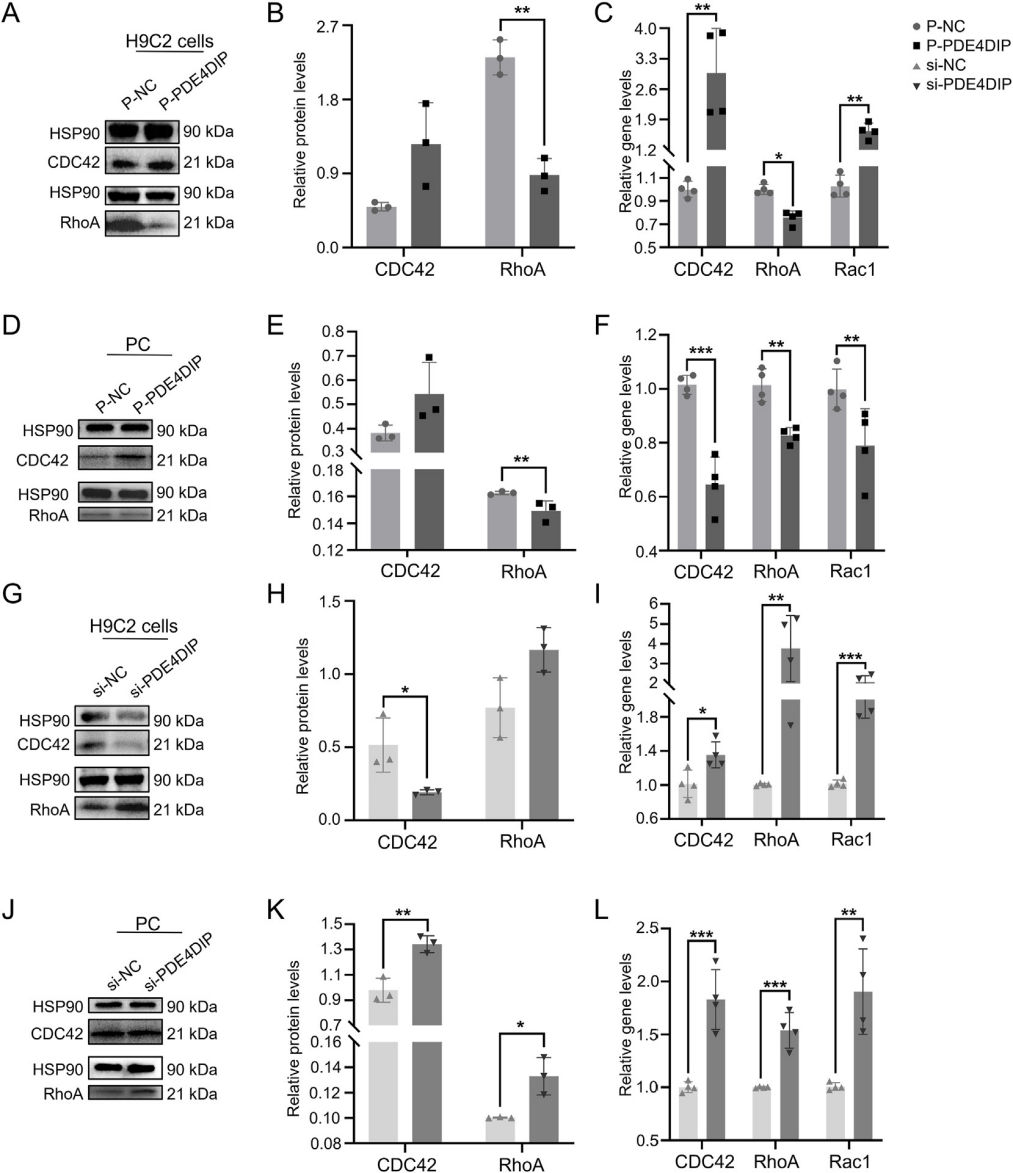
PDE4DIP plays a role in cell polarity and skeleton through the RhoA-ROCK pathway. **(A**–**C)** Protein expression in H9C2 after transfection of plasmid-PDE4DIP, including CDC42 and RhoA, analysis of the protein expression (*n* = 3 samples per group), and quantitative reverse transcription PCR analysis was employed to assess the relative mRNA expression levels of RhoA-ROCK pathway genes, including CDC42, RhoA, and Rac1, between the P-NC group and P-PDE4DIP group (*n* = 4 samples per group). **(D**–**F)** Protein expression in PC after transfection of plasmid-PDE4DIP, including CDC42 and RhoA, analysis of the protein expression (*n* = 3 samples per group), and quantitative reverse transcription PCR analysis was employed to assess the relative mRNA expression levels of RhoA-ROCK pathway genes, including CDC42, RhoA, and Rac1, between the P-NC group and P-PDE4DIP group (*n* = 4 samples per group). **(G**–**I)** Protein expression in H9C2 after transfection of siRNA-PDE4DIP, including CDC42 and RhoA, analysis of the protein expression (*n* = 3 samples/group), and quantitative reverse transcription PCR analysis of was employed to assess the relative mRNA expression levels of RhoA-ROCK pathway genes, including CDC42, RhoA, and Rac1, between the si-NC group and si-PDE4DIP group (*n* = 4 samples per group). **(J**–**L)** Protein expression in PC after transfection of siRNA-PDE4DIP, including CDC42 and RhoA, analysis of the protein expression (*n* = 3 samples per group), and quantitative reverse transcription PCR analysis was employed to assess the relative mRNA expression levels of RhoA-ROCK pathway regenes, including CDC42, RhoA, and Rac1, between the si-NC group and si-PDE4DIP group (*n* = 4 samples per group). ∗∗∗*p* < 0.001, ∗∗*p* < 0.01, and ∗*p* < 0.05 versus the NC group.

**Figure 9:**
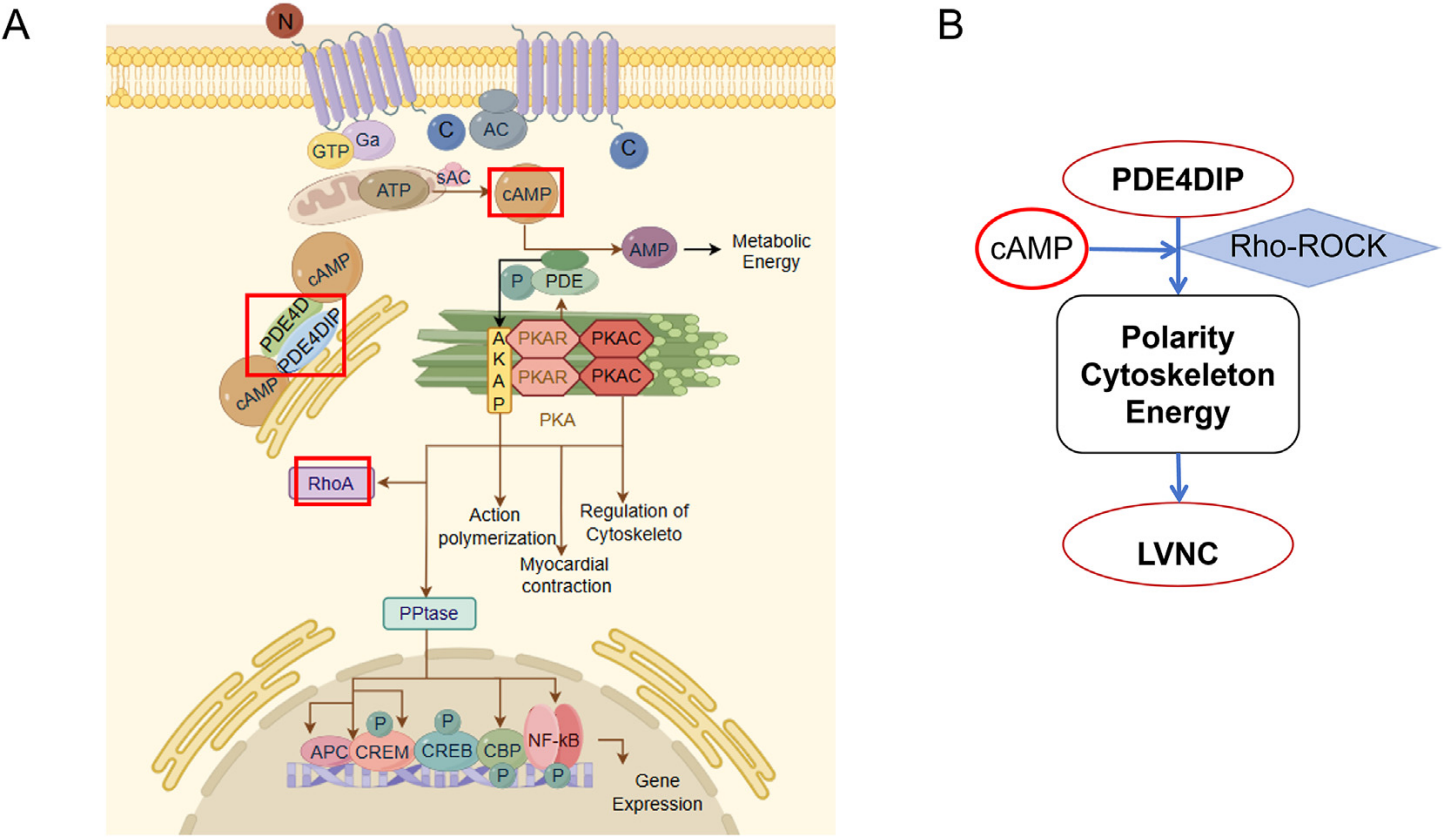
Possible mechanism of PDE4DIP causing LVNC through Rho-ROCK pathway. **(A)** PDE4DIP is located in the Golgi apparatus and regulates a series of physiological activities of cells through the Rho-ROCK pathway. **(B)** Simplified mechanism of PDE4DIP possibly causing LVNC.

Rho-ROCK pathway is a classical signal pathway in cell polarity and skeleton.^27^, ^28^, ^29^, ^30^ Overexpression of PDE4DIP in H9C2 cells and PC could reduce the expression of RhoA, one typical protein in the Rho/ROCK pathway, and promote the expression of CDC42, one upstream protein of this pathway. Gene expression of three canonical Rho GTPases and RhoA decreased, while CDC42 and Rac1 increased in H9C2 cells and decreased in PC (Fig. 8A–F). On the contrary, the knockdown of PDE4DIP could increase the expression of appeal protein and gene except for protein CDC42 in H9C2 cells (Fig. 8G–L). It was found that overexpression of PDE4DIP decreased the expression of RhoA, the most representative role of the Rho-ROCK pathway, which led to the decrease of the cell's ability to form polarity and skeleton. Knockdown of PDE4DIP would contribute to the expression of related proteins and genes in the Rho-ROCK pathway, thus contributing to the cell's polarity and skeleton. Combined with the localization and biological function of PDE4DIP in cells, the possible mechanism of PDE4DIP on cell polarity, skeleton, and energy metabolism was drawn (Fig. 9A) (the mechanism diagram was drawn using the website: https://www.figdraw.com/). These results suggested that PDE4DIP affected cell polarity, cytoskeleton, and energy metabolism through the Rho-ROCK pathway, which might lead to the cause of LVNC (Fig. 9B).

## Discussion

LVNC, as a specific type of congenital heart disease,^1^^,^^4^, ^5^, ^6^, ^7^^,^^31^^,^^32^ has been identified to show serious clinical symptoms, and limited treatment methods are available in the clinic.^9^, ^10^, ^11^ Therefore, identifying potential pathogenic targets responsible for LVNC development may provide new insights to understand the mechanisms of LVNC pathogenesis and shed light on potential therapeutic strategies against LVNC. Here, we identified that the pathogenesis of LVNC may include cell polarity, skeleton, and energy metabolism, and PDE4DIP expression was increased in the LVNC patient group compared with normal subjects. Functionally, overexpression of PDE4DIP induced cytoskeletal disorganization, decreased ATP content and cell migration, and increased cell proliferation and mitochondrial vacuolation. Moreover, the knockdown of PDE4DIP promoted cytoskeleton formation, increased ATP content, and elevated cell migration. Mechanistically, PDE4DIP overexpression inhibited the expression of polarity- and skeleton-related genes, while PDE4DIP knockdown increased these genes' expression. Meanwhile, PDE4DIP overexpression up-regulated CDC42 and Rac1 expression but reduced RhoA expression, while knockdown of PDE4DIP decreased CDC42 and Rac1 expression but increased RhoA expression, suggesting that a critical regulation of PDE4DIP to Rho-ROCK pathway. Our finding suggests that PDE4DIP activation plays a critical role in LVNC development by regulating cell polarity, skeleton, and energy metabolism via the Rho-ROCK pathway, not only providing a causal relationship of PDE4DIP activation and LVNC but also offering potential targets and therapeutic strategies against LVNC.

Our present work reveals that dysregulation of PDE4DIP is sufficient to disturb cytoskeleton formation, cell migration, cell proliferation, and mitochondrial vacuolation, providing functional evidence of PDE4DIP activation in LVNC development. Here, we first performed whole-exon sequencing using the blood samples from the normal group and LVNC patient group and found that the possible pathogenic genes were enriched in cell polarity, cytoskeleton, and mitochondria through gene ontology analysis, which may play important roles in heart development.^33^^,^^34^ Using hiPSC-CMs from three typical clinical LVNC family lines,^20^ we then identified that PDE4DIP was differentially expressed in LVNC-derived and normal control (NC)-derived hiPSC-CMs,^20^ indicating that PDE4DIP could be potential pathogenic candidate gene of LVNC. Cell polarity plays a key role in many biological processes, such as cell differentiation, cell migration, cell division, and the formation of tissues and organs.^27^^,^^35^ The cytoskeleton is an important component of the cell, mainly composed of microtubules, microfilaments, and intermediate filaments, which are closely related to physiological activities such as the maintenance of cell morphology, cellular activity, transport of signaling proteins, and cell division.^36^ In addition to this, the cytoskeleton also senses, integrates, and transmits both intracellular and extracellular signals and mediates the transport of the cellular polarity complexes to the apical/bottom end of the cells to be anchored on the cell membrane.^37^ Thus, cell polarity and the skeleton are jointly involved in the arrangement and compaction of cardiac trabeculae, as well as being closely associated with the manifestation of cardiomyocyte non-densification.^5^^,^^36^, ^37^, ^38^, ^39^ The synthesis of cAMP, an important substance in energy metabolism, activates protein kinase A (PKA), which in turn phosphorylates Ca^2+^-regulated proteins to regulate cellular energy metabolism. PKA is kept near its substrate by A-kinase anchoring protein (AKAPS), which is associated with phosphatases and phospholipases.^40^^,^^41^ PDE4DIP, as a part of the AKAP protein complex,^13^^,^^14^ is located in the Golgi apparatus of cells^16^ and is mainly expressed near the *Z* disc and sarcoplasmic reticulum in the sarcomere of the myocardium and skeletal muscle.^15^ As a member of the phosphodiesterase family, PDE4DIP can regulate the energy metabolism of cells by affecting the synthesis and degradation of cAMP.^13^ In this study, we found that overexpression of PDE4DIP induced cytoskeletal disorganization and decreased ATP content, while knockdown of PDE4DIP promoted cytoskeleton formation and increased ATP content. Moreover, we also revealed that PDE4DIP overexpression could inhibit the expression of polarity- and skeleton-related genes, while PDE4DIP knockdown increased these genes' expression. Thus, these data suggest that PDE4DIP is associated with cell polarity and cytoskeleton formation, providing functional evidence of PDE4DIP activation in LVNC development.

Our finding reveals that the Rho-ROCK pathway is involved in PDE4DIP-affected cell polarity, cytoskeleton, and energy metabolism, offering mechanistic explanations for the aberrant PDE4DIP to LVNC pathogenesis. Rho-ROCK pathway is a classical signal pathway in cell polarity and skeleton.^27^, ^28^, ^29^, ^30^ Overexpression of PDE4DIP in H9C2 cells and PC could reduce the expression of RhoA, one typical protein in the Rho/ROCK pathway, and promote the expression of CDC42, one upstream protein of this pathway. Gene expression of three canonical Rho GTPases and RhoA was decreased, while CDC42 and Rac1 were increased in H9C2 cells but decreased in PC. On the contrary, the knockdown of PDE4DIP could increase the expression of appeal protein and gene except protein CDC42 in H9C2 cells. It was found that overexpression of PDE4DIP decreased the expression of RhoA, the most representative role of the Rho-ROCK pathway, which led to the decrease of the cell's ability to form polarity and skeleton. Knockdown of PDE4DIP would contribute to the expression of related proteins and genes in the Rho-ROCK pathway, thus contributing to the cell's polarity and skeleton. Combined with the localization and biological function of PDE4DIP in cells, in terms of these results above, it is highly possible that dysregulation of PDE4DIP can cause the change of cell polarity, skeleton, and energy metabolism through the Rho-ROCK pathway, thereby contributing to LVNC pathogenesis. It still needs to be investigated in further study.

In conclusion, our findings suggest that PDE4DIP plays a critical role in LVNC development by regulating cell polarity, skeleton, and energy metabolism via the Rho-ROCK pathway, not only providing new insights to understand the LVNC pathogenesis but also offering potential targets and therapeutic strategies against LVNC.

## CRediT authorship contribution statement

**Wuxia Gu:** Writing – review & editing, Writing – original draft, Visualization, Validation, Software, Resources, Project administration, Methodology, Investigation, Formal analysis, Data curation, Conceptualization. **Hongyan Li:** Supervision, Methodology, Formal analysis, Data curation, Conceptualization. **Wenjing Yuan:** Software, Resources, Methodology, Investigation, Data curation. **Xiaoqiong Fu:** Resources, Methodology, Data curation, Conceptualization. **Rui Wang:** Visualization, Methodology, Formal analysis, Data curation. **Xiaohui Xu:** Visualization, Software, Formal analysis. **Xuemei Liao:** Visualization, Software, Formal analysis. **LingJuan Liu:** Supervision, Resources, Project administration, Formal analysis. **Bo Pan:** Supervision, Formal analysis, Conceptualization. **Jie Tian:** Supervision, Project administration, Formal analysis, Conceptualization. **Haixin Yuan:** Writing – review & editing, Supervision, Funding acquisition, Conceptualization. **Yi Huang:** Writing – review & editing, Visualization, Supervision, Conceptualization. **Tiewei Lu:** Writing – review & editing, Visualization, Project administration, Methodology, Funding acquisition, Data curation, Conceptualization.

## Ethics declaration

All procedures have been reviewed and approved by the Medical Research Ethics Committee of Children's Hospital Affiliated to Chongqing Medical University (File No: (2023) Ethics Review (Research) No. 577). All animal-related procedures were reviewed and approved by the Institutional Animal Care and Use Committee of the Affiliated Children's Hospital of Chongqing Medical University (IACUC Issue No: CHCMU-IACUC20231122013). All authors approved the manuscript and agreed with its submission to *Genes & Diseases.*

## Funding

This study was partly supported by the 10.13039/501100001809National Natural Science Foundation of China (No. 81570218, 82170244), the Program for Youth Innovation in Future Medicine of 10.13039/501100004374Chongqing Medical University (No. W0176), and the National Clinical Key Specialty Construction Project (China) (No. 010140).

## Conflict of interests

The authors declared no conflict of interests.

## Figures and Tables

**figs1:**
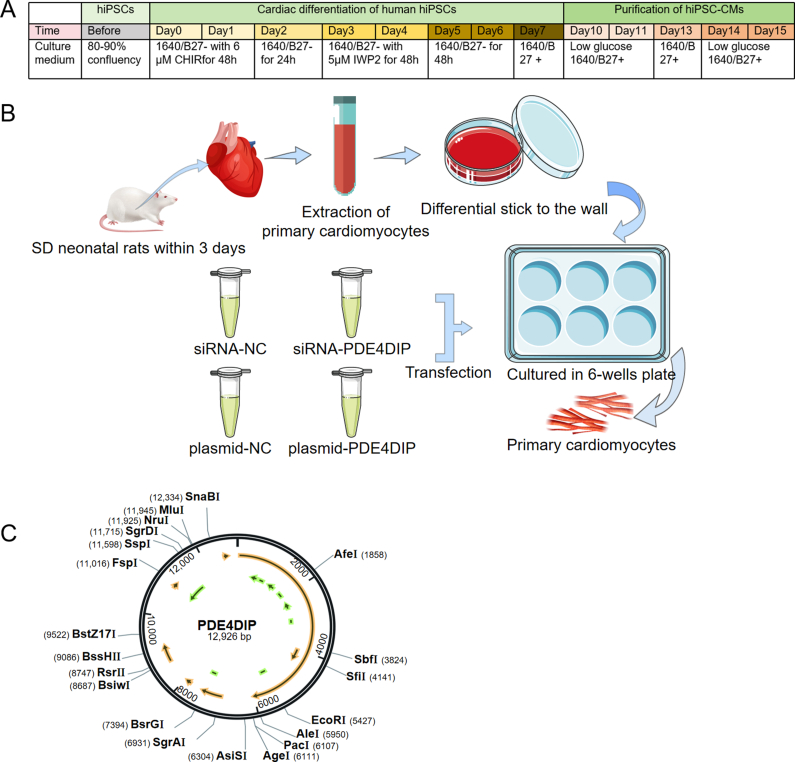


**figs2:**
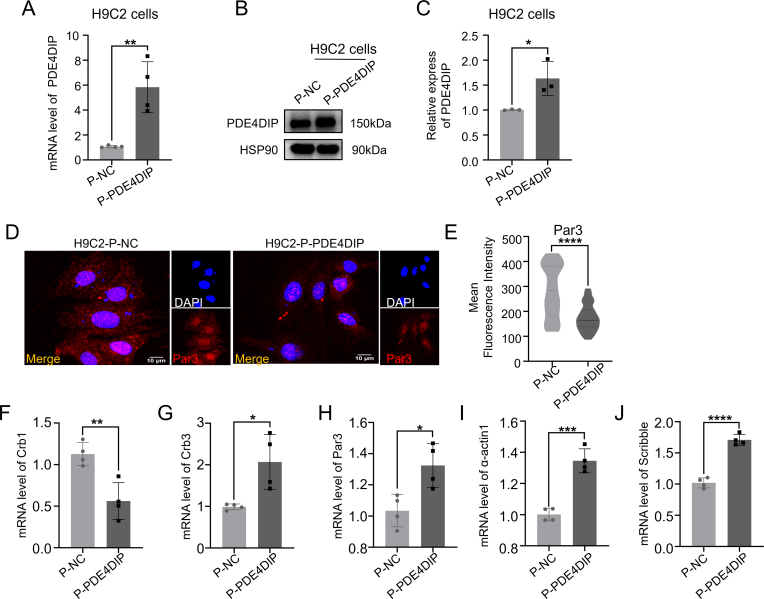


**figs3:**
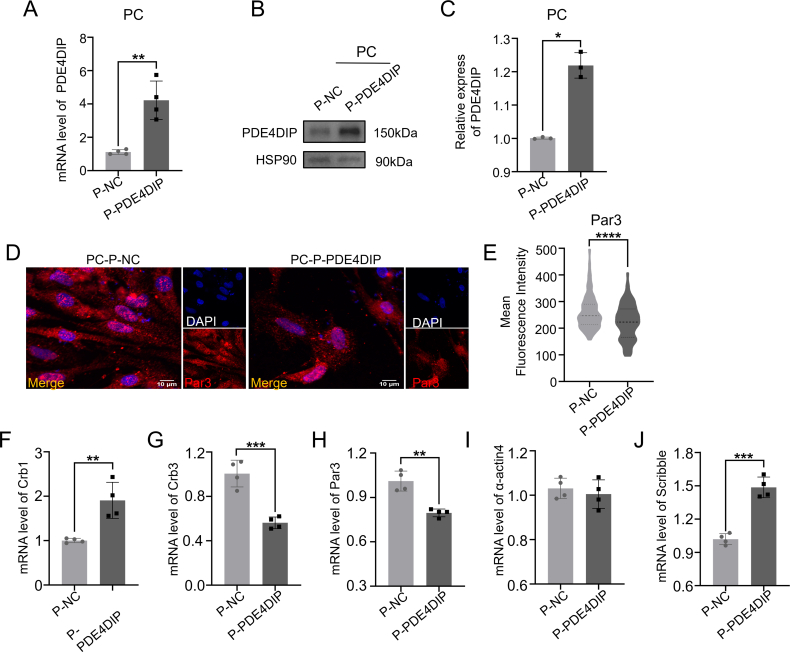


**figs4:**
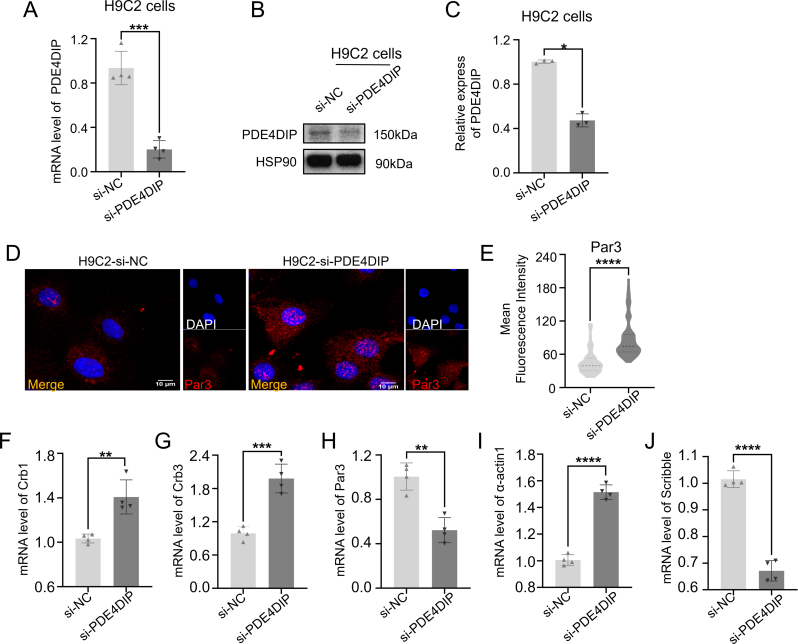


**figs5:**