# Supplementary material for: Dysfunction of PDE4DIP contributes to LVNC development by regulating cell polarity, skeleton, and energy metabolism via Rho-ROCK pathway
Source: Genes Dis. 2025 Feb 21;12(5):101568. doi: 10.1016/j.gendis.2025.101568 (PMC12221769; doi:10.1016/j.gendis.2025.101568)
Supplement: Multimedia component 1 [file mmc1.docx]

**·Supplemental material 1**: Plasmid sequencing result, with sequencing result file attached

**·Supplemental material 2**: Materials and methods

**S.2.1. Subjects and Whole-Exon Sequencing (WES)**

The whole exon library of the patients was enriched with the gDNA of peripheral blood of 3 LVNC families, and subsequently sequenced using the Illumina HiSeq 2500 sequencing system (San Diego, CA, USA). Sequencing reads were analyzed using the following workflow to identify variants in patients. Firstly, the quality of sequencing reads was checked with FastQC

(http://www.bioinformatics.babraham.ac.uk/projects/fastqc/). Duplicated reads were removed with Picard software. The remaining reads were mapped to the human reference genome (https://hgdownload.soe.ucsc.edu/downloads.html) with BWA software. The alignments were refined with tools of the GATK suite to obtain sequences that can be analyzed. Variants were called according to GATK Best Practice recommendations (https://software.broadinstitude.org/gatk/best-practices) to get the SNV/InDel loci of each sample, and the single nucleotide variants (SNV) library was established by filtering. All SNV/ InDel loci were compared and analyzed with the latest published population database, functional database, disease database and other known information, and the mutation frequency, functional characteristics, conservativeness, pathogenicity and other information of these SNV/ InDel loci were evaluated, and the corresponding possible pathogenic genes were obtained. The inter-section was taken among the LVNC possible pathogenic genes in the three families to obtain the common potential LVNC pathogenic genes^[21]^.

**S.2.2. Cell lines, reagents and animal**

Sendai virus was used to induce urine samples collected from 3 LVNC families and normal control subjects into hiPSCs. HiPSCs were cultured with PGM1 medium (CELLAPY Biological Technology, China) in Matrigel (Corning,USA)- coated 12-well cell culture plates, the cells were dissociated at about 90% confluence with EDTA (CELLAPY Biological Technology, China) and then plated in a Matrigel-coated 12-well plate.

The hiPSCs were induced to differentiate into cardiomyocytes (hiPSC-CMs) when the confluence reached 90–95%, and the undifferentiated hiPSCs were induced to differentiate into hiPSC-CMs by transient activation/inhibition of the Wnt signaling pathway. In brief, hiPSCs at 90% confluence were used for differentiation (day 0), and the medium was replaced with RPMI Medium 1640 basic (Gibco, USA) with B27 supplement minus insulin (Gibco, USA), which was considered basal medium. At day 0, cells were incubated with 6 µmol of CHIR99021 (GSK-3 inhibitor) (Selleck, USA) for 48 h. Cells were cultured in the basal medium for 24 h at 2_nd_ day. Cells were treated with 5 µmol of IWP2 (Wnt inhibitor) (Selleck, USA) for 48 h at 3_rd_ day. Then, differentiated cells were cultured in the basal medium for 48 h. On day 7, the medium was replaced by RPMI 1640 with B27 supplement with insulin (Thermo, USA). Subsequently, cells were maintained in the medium containing RPMI 1640 plus B27 supplement with insulin for 7 days. Cells were maintained in the cardiac enrichment medium [RPMI 1640 medium (Thermo, USA) supplemented with 4 mM sodium L-lactate (Sigma-Aldrich, USA)] for 3 days. After this enrichment phase, the medium was changed to RPMI 1640 with knockout serum replacement (KSR) (Thermo, USA). On day 20, spontaneously beating cardiomyocytes were dissociated with Tryple Express enzyme (Thermo, USA), centrifuged, re-suspended, and re-plated onto Matrigel-coated plates. On the day 30 of differentiation, the beating hiPSC-CMs (**Supplementary Video 1**) were used in the next experiments.

Primary cardio-myocytes were isolated from SD rats from 1 to 3 days. Hearts were rapidly excised and placed in 4°C PBS. The hearts were minced, rinsed with 0.06% Collagenase type II (Worthington, USA) solution for 1 minute. After standing for 1 minute, the supernatant was sucked to DMEM/F-12 medium containing 10% FBS serum to stop digestion. Repeat this operation until the heart tissue was completely digested. The digested cell suspension was centrifuged at 1,200 rpm for 5 minutes. The resulting pellet was suspended in medium and pre-plated for 2 hours in DMEM/F-12 containing 10% FBS to enrich the cell suspension with cardiacmyocytes by allowing attachment of fibroblasts. The unattached cells were collected and resuspended with DMEM/F-12 containing 10% FBS to obtain primary myocardial cells. The schematic diagram of extraction, culture, and transfection of primary cardiomyocytes could be seen in **s.Fig.1. A**. All cells were grown in an incubator containing 5% CO_2_ at 37℃.

**·Supplemental material 3**: Figures and videos

**Supplemental.Video 1**: hiPSC-CMs constantly spontaneous contraction when 30 days of inducement.

| LVNC-hiPSC-CMs | NC-hiPSC-CMs |
| --- | --- |
|  |  |
| 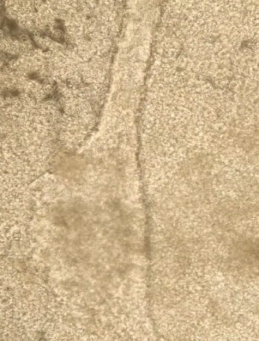 | 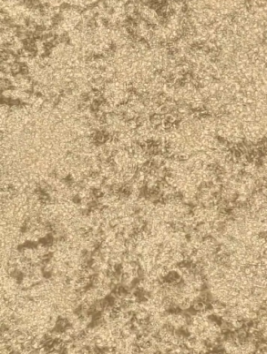 |

**Supplemental.Video 2**: Beating primary cardiomyocytes of SD neonatal rats

|  |
| --- |
| 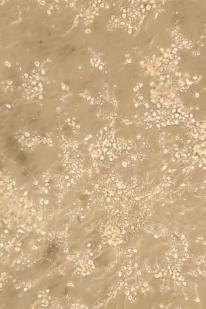 |

**Figure S1** The scheme of hiPSCs inducement, PC extraction, and plasmid structure. **(A)** Differentiation scheme of hiPSCs to hiPSCs-CMs. **(B)** Schematic diagram of extraction, culture, and transfection of primary cardiomyocytes. **(C)** Structure of the plasmid-PDE4DIP.

**
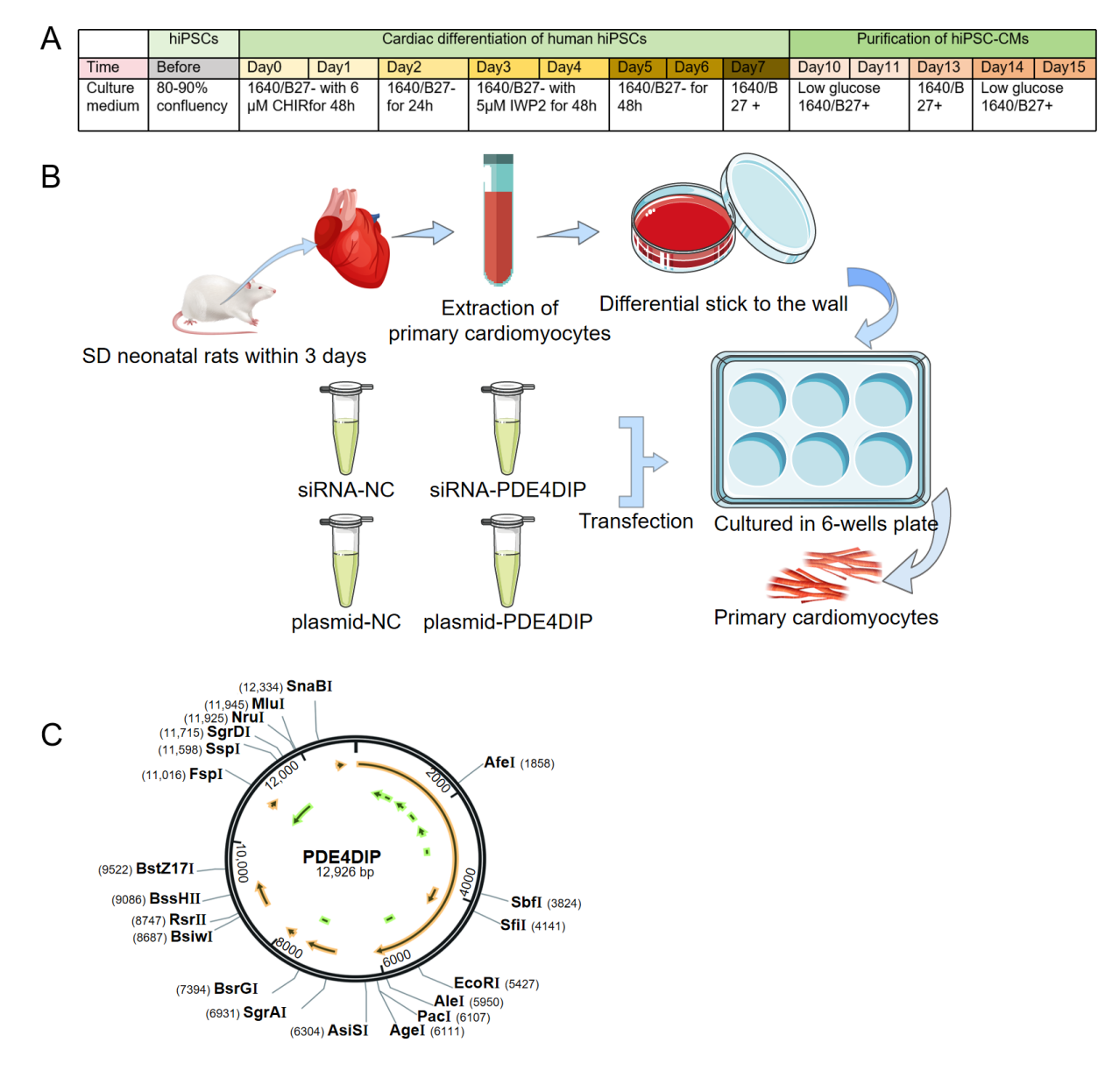
**

**Figure S2** P-PDE4DIP has abnormal skeleton and polarity in H9C2 cells, compared with the P-NC. **(A)** Quantitative reverse transcription PCR analysis of PDE4DIP relative mRNA expression after transfection of plasmid-PDE4DIP. **(B, C)** Protein expression of PDE4DIP, and analysis of the PDE4DIP protein expression (*n* = 3 samples per group). **(D, E)** Immunostaining of H9C2 cells transfected with plasmids of Par3, and analysis of the fluorescence intensity level (*n* = 40–50 cells per group). **(F–J)** Quantitative reverse transcription PCR analysis was employed to assess the relative mRNA expression levels of cell polarity and cytoskeleton genes, including Crb1, Crb3, Par3, α-actin1, and Scribble in H9C2 cells between the P-NC group and P-PDE4DIP group (*n* = 4 samples per group). ^****^*P* < 0.0001, ^***^*P* < 0.001, ^**^*P* < 0.01, and ^*^*P* < 0.05 versus the NC group.

**
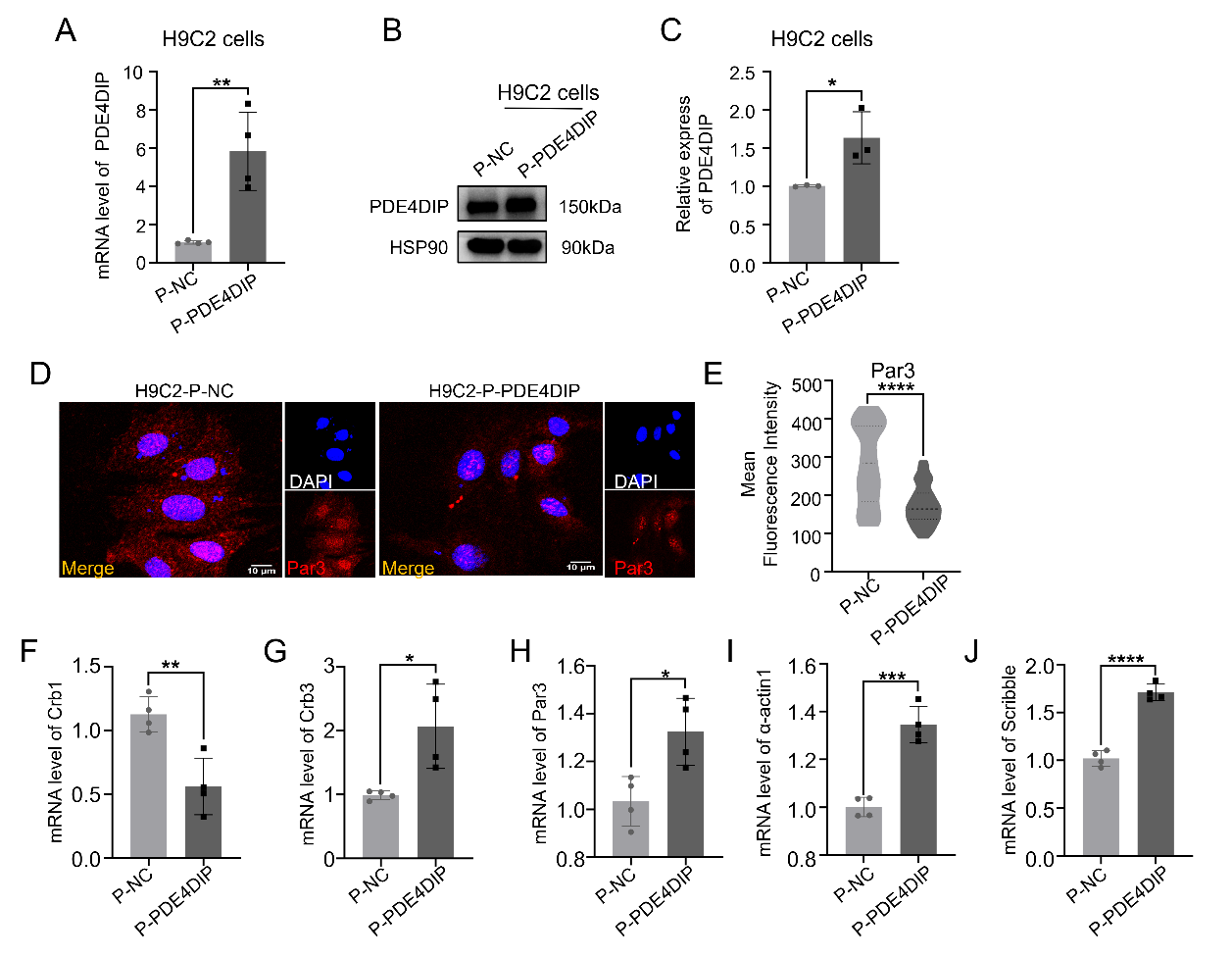
**

**Figure S3** P-PDE4DIP has abnormal skeleton and polarity in primary cardiomyocytes of neonatal Sprague-Dawley rats within 3 days of birth (PC), compared with the P-NC. **(A)** Quantitative reverse transcription PCR analysis of PDE4DIP relative mRNA expression after transfection of plasmid-PDE4DIP. **(B, C)** Protein expression of PDE4DIP, and analysis of the PDE4DIP protein expression (*n* = 3 samples per group). **(D, E)** Immunostaining of PC for Par3, and analysis of the fluorescence intensity level (*n* = 110–120 cells per group). **(F–J)** Quantitative reverse transcription PCR analysis was employed to assess the relative mRNA expression levels of cell polarity and cytoskeleton genes, including Crb1, Crb3, Par3, α-actin4, and Scribble between the P-NC group and P-PDE4DIP group (*n* = 4 samples per group). ^****^*P* < 0.0001, ^***^*P* < 0.001, ^**^*P* < 0.01, and ^*^*P* < 0.05 versus the NC group.

**
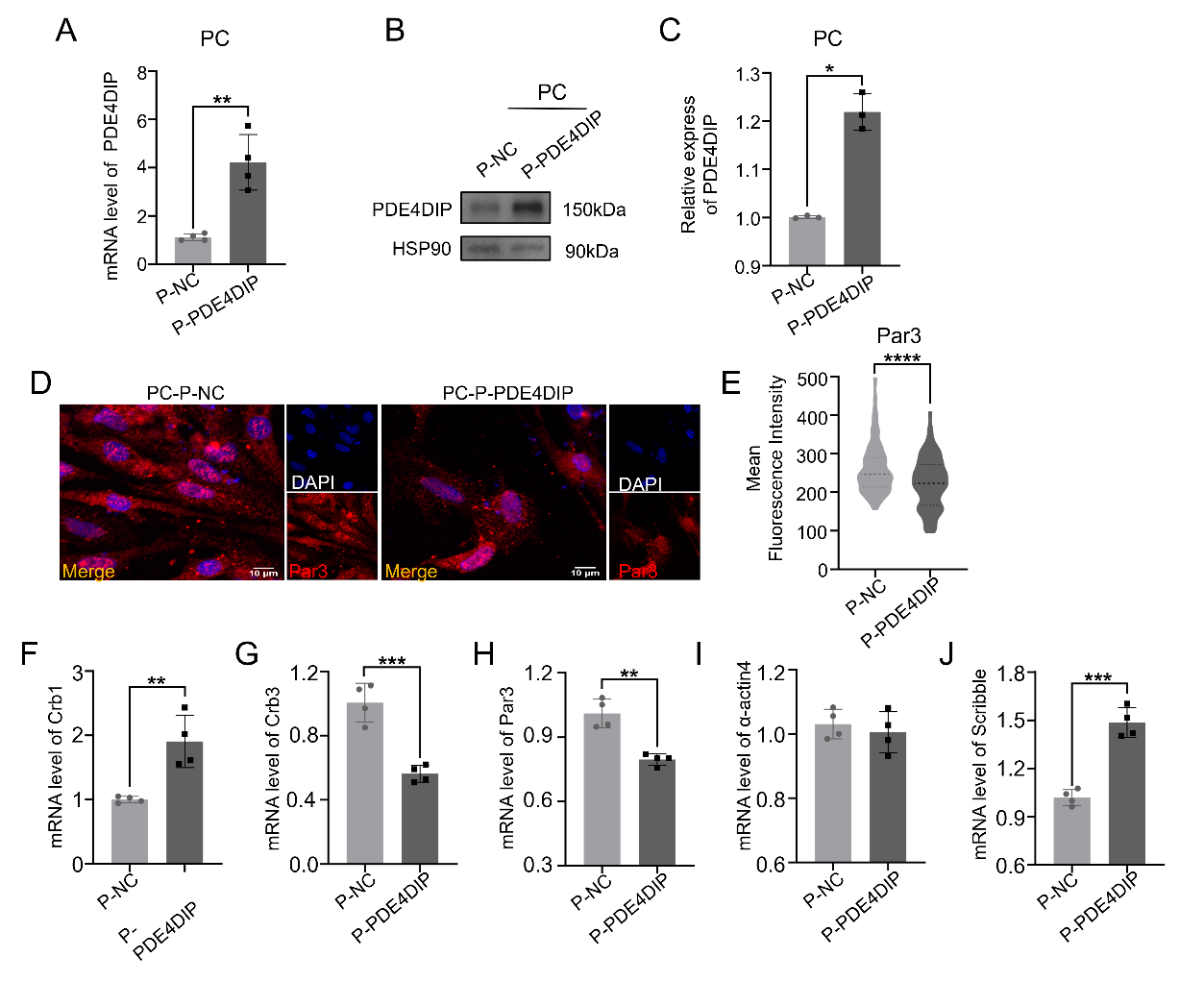
**

**Figure S4** Changes in skeleton, polarity, and mitochondria after transfection of siRNA-PDE4DIP in H9C2 cells. **(A)** Quantitative reverse transcription PCR analysis of PDE4DIP relative mRNA expression after transfection of siRNA-PDE4DIP. **(B, C)** Protein expression of PDE4DIP, and analysis of the PDE4DIP protein expression (*n* = 3 samples per group). **(D, E)** Immunostaining of H9C2 cells transfected with siRNA for Par3, and analysis of the fluorescence intensity level (*n* = 60–120 cells per group). **(F–J)** Quantitative reverse transcription PCR analysis was employed to assess the relative mRNA expression levels of cell polarity andcytoskeleton genes, including Crb1, Crb3, Par3, α-actin1, and Scribble in H9C2 cells between the si-NC group and si-PDE4DIP group (*n* = 4 samples per group). ^****^*P* < 0.0001, ^***^*P* < 0.001, ^**^*P* < 0.01, and ^*^*P* < 0.05 versus the NC group.

**
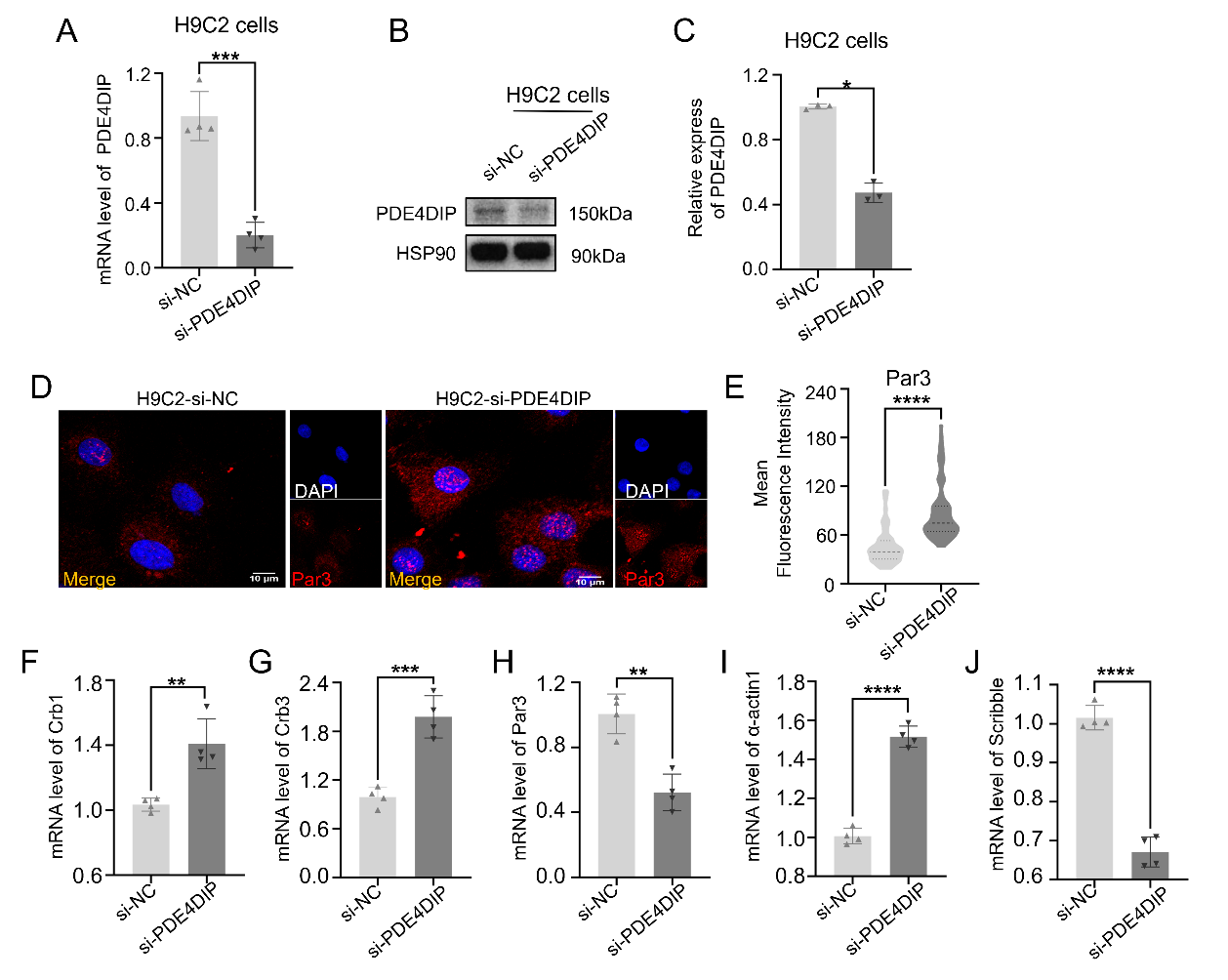
**

**Figure S5** Changes in skeleton, polarity, and mitochondria after transfection of siRNA-PDE4DIP in PC. **(A)** Quantitative reverse transcription PCR analysis of PDE4DIP relative mRNA expression after transfection of siRNA-PDE4DIP. **(B, C)** Protein expression of PDE4DIP, and analysis of the PDE4DIP protein expression (*n* = 3 samples per group). **(D, E)** Immunostaining of PC transfected with siRNA for Par3, and analysis of the fluorescence intensity level (*n* = 80–120 cells per group). **(F–J)** Quantitative reverse transcription PCR analysis was employed to assess the relative mRNA expression levels of cell polarity and cytoskeleton genes including Crb1, Crb3, Par3, α-actin4, and Scribble in H9C2 cells between the si-NC group and si-PDE4DIP group (*n* = 4 samples per group). ^****^*P* < 0.0001, ^***^*P* < 0.001, ^**^*P* < 0.01, and ^*^*P* < 0.05 versus the NC group.

**
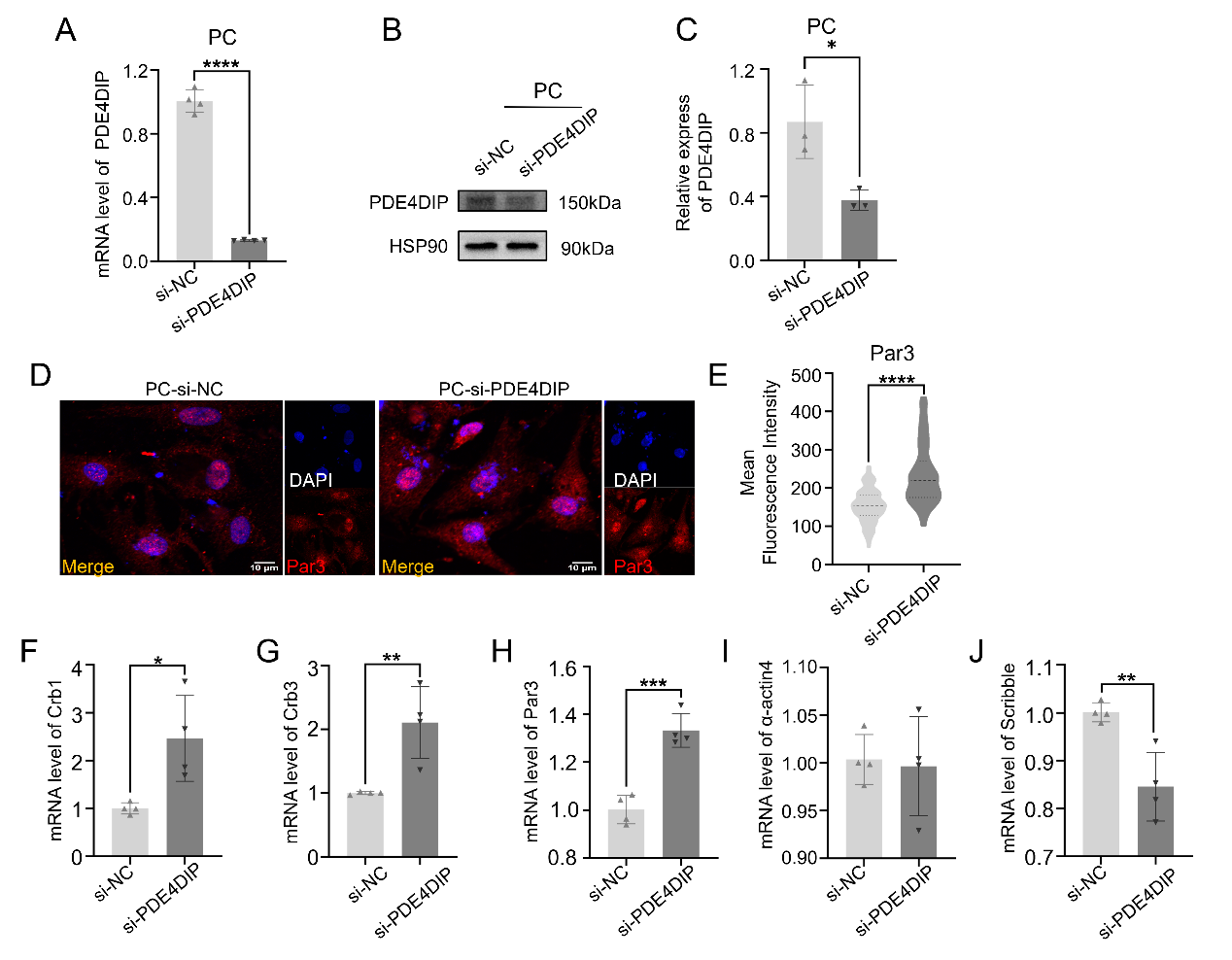
**

**Table S1** Primer information.

| Genes | Primer sequence | |
| --- | --- | --- |
|  | Forward (5' to 3') | Reverse (5' to 3') |
| rat-Myh6 | ACACCAGCCTCATCAACCAG | TGTCCTGCTCCTTCTTCAGC |
| rat-Par3 | CAGGAGCAGAGGCGAAAAGA | CTGAGATGGAGACGGGGGTA |
| rat-Filamin C | CTTTCCCTGCTGTTTTCGGC | TCTCTGCGGCTTCTGTCTTG |
| rat-Par6b | CATTGAAGGCAGTGGTGAGC | TGGAGAGGGGAGAACCCATT |
| rat-Scribble | TGTGGAGAGCAGCCAGAATG | GGTGTAGGTGATGGTGTGGG |
| rat-α-actin1 | AGCCAGGAACAGATGAACGA | GCGGTTTGGGTCTACAATGC |
| rat-α-actin4 | CGAGATGAACGGGACTCTGG | CATTTGCTCCTGGCTGATGC |
| rat-α-tubulin | CCAGATGCCAAGCGACAAGACC | GGTAGGTGCCAGTGCGAACTTC |
| rat-PDE4DIP | ACTGGTAGGCGAAGAGGGAA | TGGTCAGATGGCTCACGATG |
| rat- GAPDH | GCTCTCTGCTCCTCCCTGTT | TGGTGATGGGTTTCCCGTTG |
| rat- RhoA | GGTGATTGTTGGTGATGGAGC | GGGCACATAAACCTCTGGGA |
| rat- CDC42 | GTATGTGGAGTGTTCCGCCC | CGGCTCTTCTTCGGTTCTGG |
| rat-Rac1 | TCAGCACTCACACAGCGAG | AACAGGAGGGGGACAGAGAA |
| rat-Crb1 | AGGCAGGCATTGTGACTTGGAAG | CTCGCAGTTCACGCCAGAGTAC |
| rat-Crb2 | GCCATGCTCCAAGCCACACTC | TCACTTCCACCTGATGCCAATGC |
| rat-Crb3 | GGAGGGTTCCCAGAATGGAA | TGGGAACCCTCCTCATAGCAGTG |
| H-GAPDH | GGGAAGGTGAAGGTCGGAGT | AGCATCGCCCCACTTGATTT |
| H-PDE4DIP | GGTGGAAGCAGAGAAGGAGTG | GCCTCAGTGTATTGGTCGGG |

**Table S2** Antibody information.

|  | Antibody | Manufacturer | Dilution |
| --- | --- | --- | --- |
| Immunofluorescence staining | Anti-PDE4DIP | Solarbio | 1:50 |
|  | Anti-α/βtubulin | CST | 1:200 |
|  | Anti-F-actin | Sigma | 1:200 |
|  | Anti-vinculin | CST | 1:200 |
|  | Anti-par3 | Proteintech | 1:200 |
|  | Anti-par6 | Abcam | 1:200 |
|  | MitoTracker-Green | Beyotime | 1:5000 |
|  | CY3 anti-rabbit IgG | Servicebio | 1:200 |
|  | CY3 anti-mouse IgG | Servicebio | 1:200 |
| Western blotting | Anti-PDE4DIP | Abcam | 1:500 |
|  | Anti-Scribble | Proteintech | 1:2000 |
|  | Anti-HSP90 | Origene | 1:5000 |
|  | Anti-RhoA | Abcam | 1:2000 |
|  | Anti-CDC42 | Abcam | 1:2000 |
|  | Anti-Rac1 | CST | 1:1000 |
|  | Anti-α/βtubulin | CST | 1:1000 |
|  | Anti-Vinculin | Sigma-Aldrich | 1:2000 |
|  | Anti-Crb2 | Abcam | 1:2000 |
|  | Anti-rabbit IgG | ZEN-BIOSCIENCE | 1:5000 |
|  | Anti-mouse IgG | Servicebio | 1:5000 |
